# Molecular profiling of aged neural progenitors identifies *Dbx2* as a candidate regulator of age‐associated neurogenic decline

**DOI:** 10.1111/acel.12745

**Published:** 2018-03-05

**Authors:** Giuseppe Lupo, Paola S. Nisi, Pilar Esteve, Yu‐Lee Paul, Clara Lopes Novo, Ben Sidders, Muhammad A. Khan, Stefano Biagioni, Hai‐Kun Liu, Paola Bovolenta, Emanuele Cacci, Peter J. Rugg‐Gunn

**Affiliations:** ^1^ Department of Chemistry Sapienza University of Rome Rome Italy; ^2^ Department of Biology and Biotechnology “C. Darwin” Sapienza University of Rome Rome Italy; ^3^ Centro de Biologia Molecular “Severo Ochoa” Consejo Superior de Investigaciones Cientificas‐Universidad Autonoma de Madrid Madrid Spain; ^4^ CIBER of Rare Diseases ISCIII Madrid Spain; ^5^ Epigenetics Programme The Babraham Institute Cambridge UK; ^6^ Bioscience Oncology IMED Biotech Unit AstraZeneca Cambridge UK; ^7^ Division of Molecular Neurogenetics German Cancer Research Centre (DKFZ) DKFZ‐ZMBH Alliance Heidelberg Germany; ^8^ Wellcome Trust – Medical Research Council Cambridge Stem Cell Institute University of Cambridge Cambridge UK

**Keywords:** DNA methylation, epigenetics, histone methylation, neural stem/progenitor cells, neurospheres, subventricular zone

## Abstract

Adult neurogenesis declines with aging due to the depletion and functional impairment of neural stem/progenitor cells (NSPCs). An improved understanding of the underlying mechanisms that drive age‐associated neurogenic deficiency could lead to the development of strategies to alleviate cognitive impairment and facilitate neuroregeneration. An essential step towards this aim is to investigate the molecular changes that occur in NSPC aging on a genomewide scale. In this study, we compare the transcriptional, histone methylation and DNA methylation signatures of NSPCs derived from the subventricular zone (SVZ) of young adult (3 months old) and aged (18 months old) mice. Surprisingly, the transcriptional and epigenomic profiles of SVZ‐derived NSPCs are largely unchanged in aged cells. Despite the global similarities, we detect robust age‐dependent changes at several hundred genes and regulatory elements, thereby identifying putative regulators of neurogenic decline. Within this list, the homeobox gene *Dbx2* is upregulated in vitro and in vivo, and its promoter region has altered histone and DNA methylation levels, in aged NSPCs. Using functional in vitro assays, we show that elevated *Dbx2* expression in young adult NSPCs promotes age‐related phenotypes, including the reduced proliferation of NSPC cultures and the altered transcript levels of age‐associated regulators of NSPC proliferation and differentiation. Depleting *Dbx2* in aged NSPCs caused the reverse gene expression changes. Taken together, these results provide new insights into the molecular programmes that are affected during mouse NSPC aging, and uncover a new functional role for *Dbx2* in promoting age‐related neurogenic decline.

## 1 INTRODUCTION

In the adult mouse forebrain, neurogenesis persists in two restricted niches located in the subventricular zone (SVZ) close to the lateral ventricles and in the subgranular zone (SGZ) within the dentate gyrus. In these regions, neural stem/progenitor cells (NSPCs) are maintained throughout adulthood and give rise to daughter cells that undergo differentiation into neurons and glia. Increasing evidence supports a role for adult neurogenesis in normal brain function and suggests that neurological disorders and neurodegeneration may be caused, at least in part, by reduced neuronal output of adult NSPCs (Goncalves, Schafer & Gage, 2016; Lledo & Valley, 2016).

Aging is a physiological process substantially affecting, in a time‐dependent manner, the function of somatic stem cells in multiple tissues (Signer & Morrison, 2013). Aging is associated with reduced neurogenesis in the mouse SVZ and SGZ (Encinas et al., 2011; Enwere et al., 2004; Lugert et al., 2010; Luo, Daniels, Lennington, Notti & Conover, 2006), which might lead to decreased olfactory function and cognitive hippocampus‐dependent impairment (Goncalves et al., 2016; Lledo & Valley, 2016). This age‐associated neurogenic decline appears to be caused both by a depletion in the NSPC pool of the aged niche (Ahlenius, Visan, Kokaia, Lindvall & Kokaia, 2009; Bouab, Paliouras, Aumont, Forest‐Berard & Fernandes, 2011; Corenblum et al., 2016; Enwere et al., 2004; Luo et al., 2006; Maslov, Barone, Plunkett & Pruitt, 2004; Molofsky et al., 2006; Stoll et al., 2011) and by the decreased capacity of the remaining NSPCs to sustain proliferation and neuronal differentiation, as revealed by in vitro studies (Ahlenius et al., 2009; Apostolopoulou et al., 2017; Corenblum et al., 2016; Daynac, Morizur, Chicheportiche, Mouthon & Boussin, 2016; Daynac et al., 2014; L'Episcopo et al., 2013; Shi et al., 2017; Zhu et al., 2014). NSPCs undergo cell autonomous age‐related changes that affect intracellular molecular pathways, including the altered expression of telomerase and cell cycle regulators, which have been linked to the decline in NSPC proliferation upon aging (Caporaso, Lim, Alvarez‐Buylla & Chao, 2003; Molofsky et al., 2006; Nishino, Kim, Chada & Morrison, 2008). Transcriptional analysis of the aged whole SVZ cell population (including NSPCs, differentiated cells and non‐neural cell types) identified several misregulated genes that are associated with NSPC proliferation and differentiation, suggesting that intrinsic gene expression changes in aged NSPCs can alter adult neurogenesis (Apostolopoulou et al., 2017; Shi et al., 2017). The transcriptional regulators that cause these effects and their roles in relaying extrinsic niche signals remain largely unclear.

Attenuating the age‐associated decline in somatic stem cell function can be achieved by modulating the extracellular environment (Adler et al., 2007; Conboy et al., 2005; Villeda et al., 2011), which implicates epigenetic regulation as a key component of stem cell aging (Beerman & Rossi, 2015; O'Sullivan & Karlseder, 2012; Rando & Chang, 2012). To date, the epigenomes of three stem cell populations have been examined upon mouse and human aging: blood stem cells, mesenchymal stem cells and muscle satellite cells (Beerman et al., 2013; Bocker et al., 2011; Bork et al., 2010; Fernandez et al., 2015; Liu et al., 2013; Sun et al., 2014). Together, these studies conclude that the stem cell epigenome is relatively stable during aging, with a small number of potentially important loci that are significantly altered (Beerman & Rossi, 2015). Genes encoding self‐renewal and differentiation factors are particularly vulnerable to age‐dependent alterations, and, although often do not have an immediate impact on transcriptional changes; they might alter the potential or future decisions of the stem cells (Beerman & Rossi, 2015). Notably, the global epigenetic profiles have not been examined in aging NSPCs or in any aged somatic stem cell from nonmesoderm derivatives. This knowledge gap is important to address to identify potential age‐associated drivers of neurogenic decline, and to understand the commonalities in intrinsic mechanisms that might underpin somatic stem cell aging.

To address these deficits, we have generated molecular profiles of NSPCs derived from the SVZ of young adult and aging mice. We identified age‐dependent changes at several hundred genes and regulatory elements, thereby identifying putative regulators of neurogenic decline. Among them, we focused our attention on the transcription factor‐encoding gene *Dbx2*, which was upregulated in aged NSPCs and has not been associated previously with aging. Functional assays revealed that increased *Dbx2* transcript levels in young adult NSPCs promote age‐related phenotypes, including the reduced proliferation of NSPC cultures and the altered expression levels of age‐associated regulators of NSPC proliferation and differentiation. Partial reduction of *Dbx2* levels in aged NSPCs caused an opposite transcriptional response. Taken together, these results provide new insights into the molecular programmes that are affected during mouse NSPC aging, and identify a *Dbx2*‐dependent transcriptional programme involved in the functional decline of NSPCs in the aged SVZ.

## 2 RESULTS

### 2.1 Comparative transcriptomic analysis of NSPCs derived from the young adult and aged SVZ

**Figure 1:**
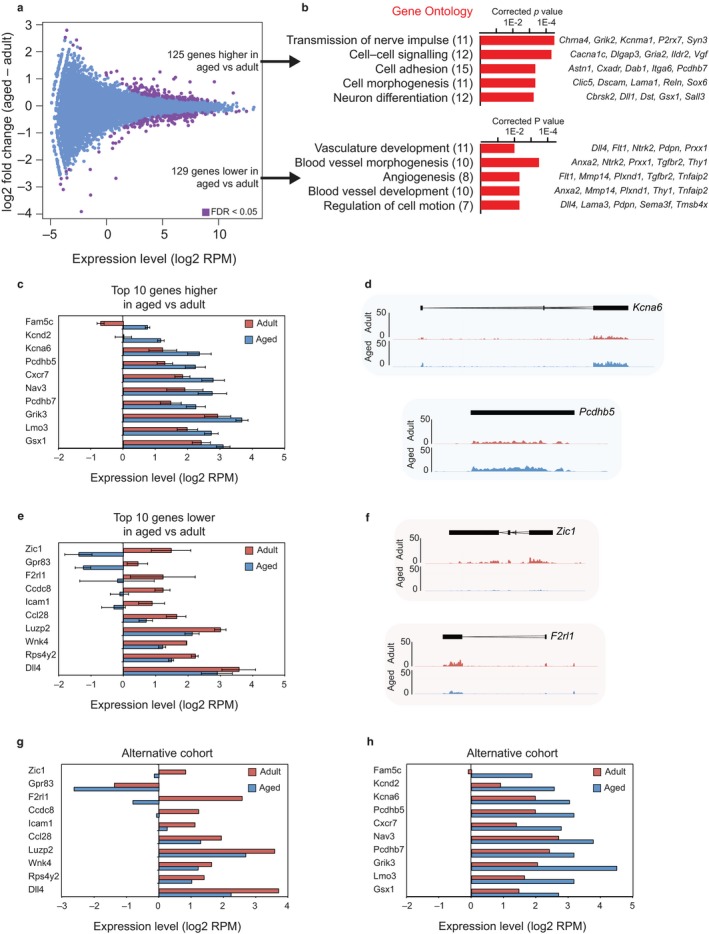
Identification of differentially expressed genes in NSPCs derived from the SVZ of young adult and aged mice. (a) MA plot of RNA‐seq data from NSPCs derived from the SVZ of aged and young adult mice. For each age, three independent derivations of SVZ NSPCs were used for this analysis. A Limma moderated *t* test with multiple testing corrections identified genes with a false discovery rate (FDR) of <.05, and these genes were classified as differentially expressed. (b) Top GO terms of differentially expressed gene sets that are upregulated (upper) and downregulated (lower) in aged compared to young adult NSPCs. Numbers of genes are shown; example genes within each GO category are listed (right). Corrected *p*‐values were calculated using a modified Fisher's exact test followed by Bonferroni's multiple comparison test. (c–f) Summary of the ten most strongly upregulated (c, d) or downregulated (e, f) genes in NSPCs from aged mice. Charts in (c, e) show log_2_ RPM expression levels in adult and aged NSPCs. Data show mean ± *SD*;* n* = 3 biological replicates. Genome browser representations in (d, f) show the density of RNA‐seq reads across transcribed regions of representative genes. (g–h) RNA‐seq analysis of an alternative set of NSPC derivations from mice that were housed in a separate facility and from a different strain (C57BL/10). The results show consistent gene expression changes for upregulated (g) and downregulated (h) genes, demonstrating that the observed age‐related transcriptional changes were independent of the genetic background and laboratory conditions of the mice

We derived three cohorts of NSPCs from the SVZ of young adult (3 months old, 3 months) and aged (18 months) mice and expanded the cells for two passages (2–3 weeks) in nonadherent culture conditions. We used neurospheres that formed during the second passage to generate expression profiles by RNA sequencing (RNA‐seq). Analysis of these data revealed that the majority of genes were expressed at similar levels between adult and aged samples, although 254 genes were differentially expressed (Figure 1a and Table S1; False Discovery Rate (FDR) < .05). As an initial confirmation of our dataset, we detected changes in *Dlx2* and *Let‐7b* expression, which are misregulated in aged NSPCs (Nishino et al., 2008; Shi et al., 2017). Furthermore, many cell type‐specific genes were detected at comparable levels in adult and aged samples, indicating that the cellular composition of the neurospheres was unchanged with age (Figure S1A).

To gain insight into the biological function of the identified genes, we performed Gene Ontology (GO) analysis of the 125 genes that were upregulated in the aged NSPCs compared with the young adult NSPCs. This analysis revealed an enrichment for processes associated with neuron differentiation, cell signalling and cell morphogenesis (Figure 1b, top). GO terms associated with the 129 genes that were downregulated in the aged NSPCs included vasculature development, angiogenesis and regulation of cell motion (Figure 1b, bottom). Misregulated genes with the largest fold change are shown in Figure 1c–f. We validated our findings with a separate cohort of adult and aged NSPCs from mice that were housed in a different facility, thereby affirming that aging itself was the main driver of the observed gene expression changes (Figure 1g,h). These results provide a robust and comprehensive gene expression data set for young adult and aged NSPCs.

### 2.2 Epigenome profiling identifies differences between young adult and aged SVZ NSPCs

Epigenetic control of gene regulation through DNA and histone modifications has a pivotal role to ensure the appropriate transcriptional programmes during embryonic and adult neurogenesis (Cacci, Negri, Biagioni & Lupo, 2017) and can be misregulated in aged stem cell compartments (Beerman & Rossi, 2015). Altered epigenetic mechanisms could also be involved in age‐associated neurogenic decline, but little is known about the epigenetic changes that occur during the aging of adult NSPCs.

**Figure 2:**
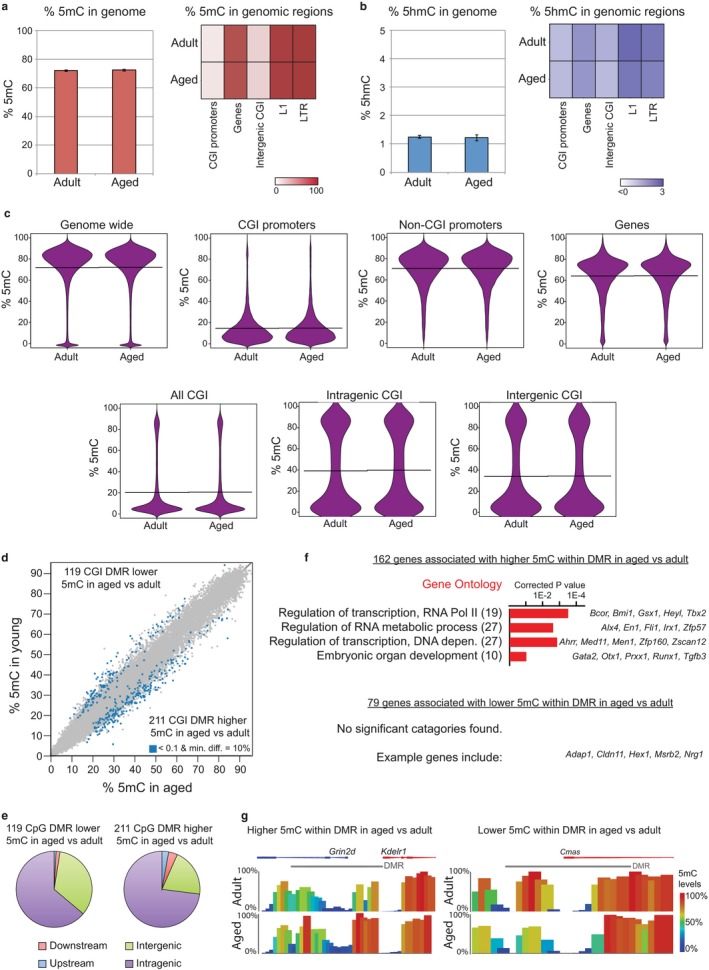
Genomewide profiling of DNA methylation in young adult and aged NSPCs identifies differentially methylated regions that are associated with gene promoters. (a–b) BS‐seq and oxBS‐seq analysis of 5mC (a) and 5hmC (b) in adult and aged NSPCs. Boxplots show the average levels of 5mC and 5hmC over the whole genome (mean ± *SD*;* n* = 3 biological replicates). Heatmaps show the percentage of 5mC and 5hmC in genes, CGIs located within promoters or intergenic regions, and transposon elements (L1, LTR). (c) Bean plots showing the distribution of 5mC levels for different genomic features in adult and aged NSPCs. (d) Scatter plot comparing 5mC levels at individual CGIs between adult and aged NSPCs. CGIs that correspond to differentially methylated regions (DMRs) are highlighted in blue (*p* < .05, chi‐squared test with multiple testing corrections, and a minimum difference in methylation of 10%). (e) Pie charts showing the percentages of DMRs that are located within less than 2 kb upstream or downstream of a gene, or that are found in intragenic or intergenic regions. (f) Top GO terms of genes that are associated with differentially methylated regions either hypomethylated (upper) or hypermethylated (lower) in aged compared to adult NSPCs. Numbers of genes are shown; example genes within each GO category are listed (right). Corrected *p*‐values were calculated using a modified Fisher's exact test followed by Bonferroni's multiple comparison test. (g) Genome browser tracks of 5mC levels in adult and aged NSPCs across representative differentially methylated CGIs (indicated by grey line) that overlap with gene promoters

We first investigated DNA methylation levels by profiling 5‐methylcytosine (5mC) and 5‐hydroxymethylcytosine (5hmC) using whole‐genome bisulphite (BS‐seq) and oxidative bisulphite (oxBS‐seq) sequencing. Globally, the levels of 5mC and 5hmC were very similar between the young adult and aged NSPCs (Figure 2a,b). In keeping with other mammalian cell types, low 5mC levels were found in CpG islands (CGIs), and high methylation levels were found in gene bodies and repetitive elements such as L1 and LTR (Figure 2a). Furthermore, the distribution of 5mC across various genome compartments was indistinguishable between the adult and aged samples (Figure 2c). To look for localized differences in DNA methylation between adult and aged NSPCs, we applied a chi‐squared test with multiple testing corrections to define differentially methylated regions (DMRs; *p*_adj_ < .05). Using this approach, we identified 330 DMRs that overlapped with CGIs (Figure 2d and Table S2). There were 119 DMRs with reduced 5mC levels (hypo‐DMRs) and 211 DMRs with increased 5mC levels (hyper‐DMRs) in the aged NSPCs compared with young adult NSPCs. The majority of hypo‐DMRs (67%) and hyper‐DMRs (80%) were within 2kb of a gene (Figure 2e) and were associated with 79 and 162 genes, respectively (Figure 2f).

GO analysis of the genes with hyper‐DMRs revealed an enrichment for biological processes associated with transcription factors including *Bmi1*,* Prdm1*,* Tbx2* and with multicellular organism development (Figure 2f). Genes associated with hypo‐DMRs were not significantly enriched for any GO categories, but example genes include *Arx, Dlg2* and *Slmo1*. Genome browser representations are shown for DMRs associated with *Grin2d* and *Cmas* (Figure 2g). Overall, there was no correlation between the changes in DNA methylation and the changes in the expression of the nearest gene (not shown), although there were several examples where the gain of DNA methylation was associated with a transcriptional downregulation upon aging (e.g. *Chl1, Zfp536*) and where the loss of DNA methylation was associated with a transcriptional upregulation (e.g. *Fam179a, Tmcc3*). These findings show that DNA methylation levels and distribution are very similar between adult and aged NSPCs, although we have identified several hundred regions that are differentially methylated and are near to genes.

**Figure 3:**
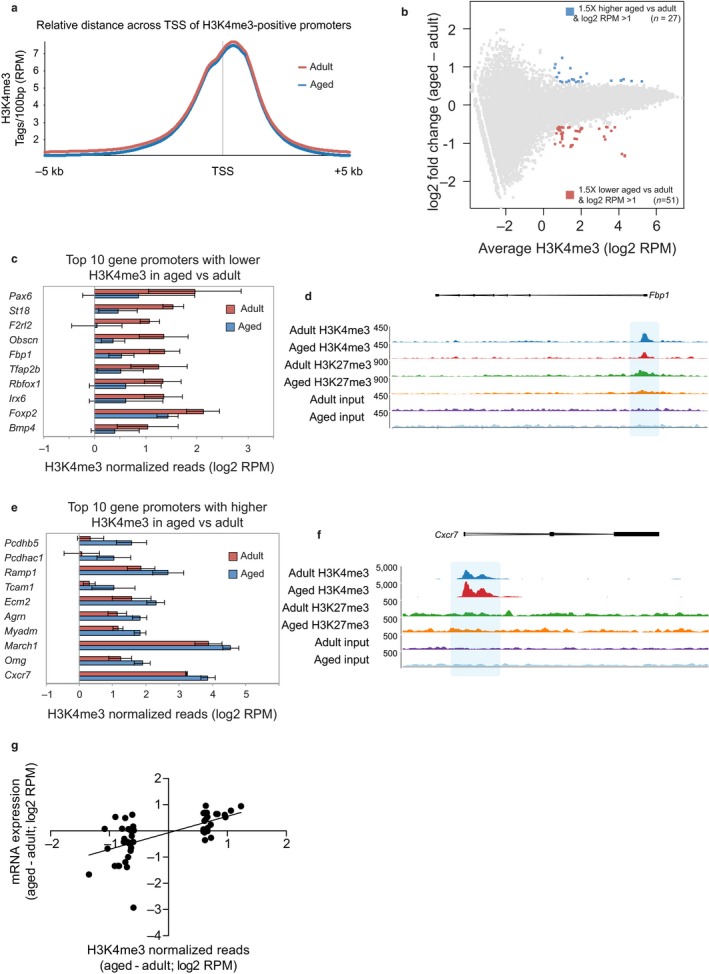
Genomewide analysis of H3K4me3 levels in young adult and aged NSPCs. (a) Quantitative trend plot of H3K4me3 normalized ChIP‐seq reads over TSS ± 5 kb. (b) MA plot of H3K4me3 ChIP‐seq data for adult and aged NSPCs. Data are shown as the average of three biological replicates. (c–f) Summary of the ten gene promoters showing the strongest decrease (c, d) or increase (e, f) in H3K4me3 signal in aged NSPCs compared to adult NSPCs. Charts in (c, e) show H3K4me3 normalized reads (log_2_ RPM) in adult and aged NSPCs. Data show mean ± *SD*;* n* = 3 biological replicates. Genome browser representations in (d, f) show the density of H3K4me3 ChIP‐seq reads across two example genes. (g) Comparison of RNA‐seq log_2_ fold change (*y*‐axis) and H3K4me3 ChIP‐seq log_2_ fold change (*x*‐axis) for the 78 transcripts that have differential H3K4me3 promoter levels between adult and aged NSPCs. There is a significant correlation between gene expression changes and differences in H3K4me3 levels according to Pearson correlation test (*p* < .0001)

We also profiled the genomewide localization of histone H3 lysine 4 trimethylation (H3K4me3) and histone H3 lysine 27 trimethylation (H3K27me3) using chromatin immunoprecipitation combined with sequencing (ChIP‐seq). We identified 30,406 H3K4me3 peaks (MACS, *p* < 1E−9) that were associated with 13,030 transcriptional start sites (TSS) in adult NSPCs, and 28,854 peaks associated with 12,955 TSS in aged NSPCs. The majority of H3K4me3 signal was centred on the TSS of genes, and this distribution profile was identical for the adult and aged NSPCs (Figure 3a). We did not observe the broadening of H3K4me3 peaks that have been reported in aged blood stem cells (Sun et al., 2014). Focusing on gene promoters (−2 kb to +0.5 kb from TSS), we identified 27 regions with increased H3K4me3 levels in the aged NSPCs compared with the adult NSPCs, and 51 regions with the opposite trend (Figure 3b and Table S3). Gene promoters with the largest changes in H3K4me3 upon NSPC aging are shown in Figure 3c–f. Overall, there was a significant correlation between the changes in H3K4me3 promoter levels and the changes in the expression of the associated gene (Figure 3g; Pearson correlation test, *p* < .0001).

**Figure 4:**
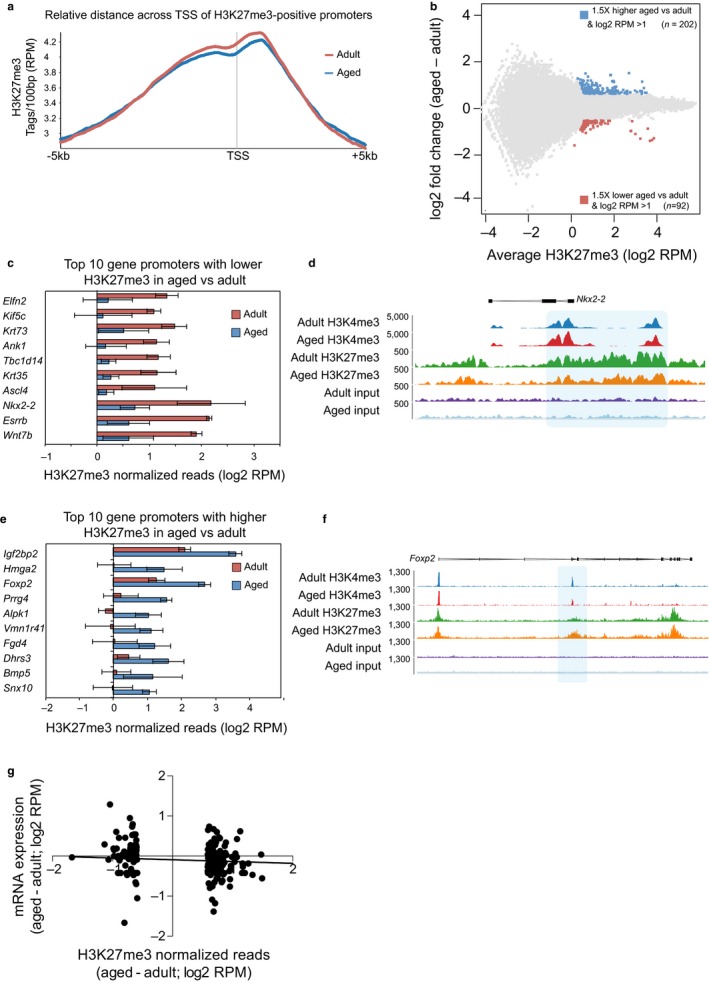
Genomewide analysis of H3K27me3 levels in young adult and aged NSPCs. (a) Quantitative trend plot of H3K27me3 normalized ChIP‐seq reads over TSS ± 5 kb. (b) MA plot of H3K27me3 ChIP‐seq data for adult and aged NSPCs. Data are shown as the average of three biological replicates. (c–f) Summary of the ten gene promoters showing the strongest decrease (c, d) or increase (e, f) in H3K27me3 signal in aged compared to adult NSPCs. Charts in (c, e) show H3K27me3 normalized reads (log_2_ RPM) in adult and aged NSPCs. Data show mean ± *SD*;* n* = 3 biological replicates. Genome browser representations in (d, f) show the density of H3K27me3 ChIP‐seq reads across two example genes. (g) Comparison of RNA‐seq log_2_ fold change (*y*‐axis) and H3K27me3 ChIP‐seq log_2_ fold change (*x*‐axis) for the 294 transcripts that have differential H3K27me3 promoter levels between adult and aged NSPCs. No significant correlation between gene expression changes and differences in H3K27me3 levels is present according to Pearson correlation test (*p* < .18)

Analysis of the H3K27me3 ChIP‐seq data identified 43,640 peaks (MACS, *p* < 1E−9) that were associated with 3,819 TSS in adult NSPCs, and 43,569 peaks associated with 3,836 TSS in aged NSPCs. The distribution of H3K27me3 across gene promoter regions was similar for the adult and aged NSPCs, with a broad peak centred on the TSS of genes (Figure 4a). Quantitation of these regions identified a set of 202 gene promoters with increased H3K27me3 levels in the aged NSPCs compared with the adult NSPCs, and 92 gene promoters with the opposite pattern (Figure 4b and Table S4). Gene promoters with the largest change in H3K27me3 upon NSPC aging are shown in Figure 4c–f. There was no correlation between the changes in H3K27me3 promoter levels and the changes in the expression of the associated gene (Figure 4g; Pearson correlation test, *p* = .18), although there were several examples where the gain of H3K27me3 was associated with a transcriptional downregulation (*Igf2bp2*,* Prrg4*,* Trp73*) and also for the converse situation (*Tnfrsf13b*,* Laptm5*,* Rhcg*).

Our results suggest that the transcriptional and epigenetic signatures of SVZ NSPCs are remarkably well preserved during aging. Nonetheless, we identified loci with significant gene expression and epigenetic differences between young adult and aged NSPCs, suggesting that the functional impairment of aged NSPCs might be caused by the altered regulation of a limited set of influential genes.

### 2.3 Identification of *Dbx2* as a candidate gene underlying age‐dependent decline of SVZ NSPCs

As an initial step towards defining the molecular modifiers of neurogenic decline, we mined our data set for genes that might play an instructive role in the altered proliferation and/or differentiation of aged NSPCs. First, we identified the genes that showed consistent transcriptional and epigenetic changes between adult and aged samples (Table S5). Second, we used qRT‐PCR to screen for genes that maintained an expression difference between adult and aged NSPCs after prolonged expansion in vitro. Third, we focused on genes showing expression changes when NSPCs were switched from proliferating to differentiating conditions. Fourth, we verified the expression of candidate genes in the adult SVZ in vivo either according to previous literature or by experimental analysis.

**Figure 5:**
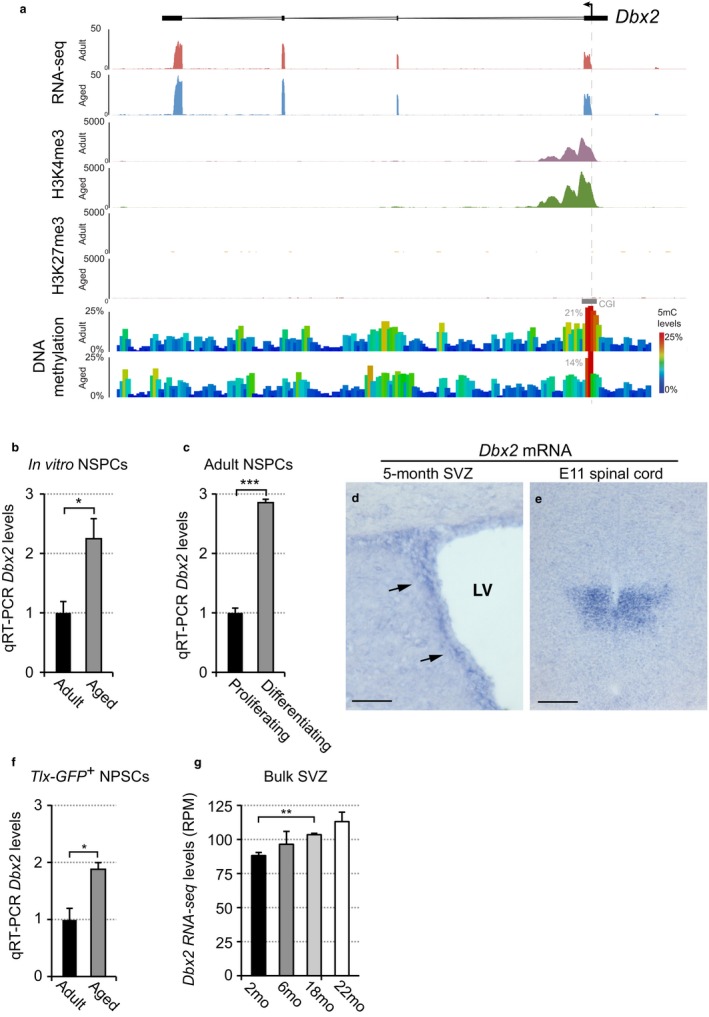
Identification of *Dbx2* as a candidate gene implicated in SVZ NSPC aging. (a) Genomewide data track for the *Dbx2* locus in young adult and aged NSPCs. The dashed line indicates the TSS; the solid grey line indicates the position of the DMR, and the percentage 5mC levels across the DMR are shown. Data represent the average of three biological replicates. Genomic coordinates for the region shown are as follows: chr15:95619911‐95661537 (GRCm38). (b) qRT‐PCR analysis of *Dbx2* expression in nonadherent cultures of adult or aged NSPCs that were expanded for 5–6 passages in vitro from dissected SVZ tissues. Data show mean ± *SEM* following normalization to adult NSPC samples; *n* = 3 biological replicates. *p < .05, Student's *t* test. (c) qRT‐PCR analysis of *Dbx2* expression in adherent cultures of adult SVZ NSPCs that were maintained in proliferating conditions or cultured for 24 hr in differentiation conditions devoid of EGF. Data show mean ± *SEM* following normalization to proliferating NSPC samples; *n* = 4 biological replicates. ***p < .001, Student's *t* test. (d, e) Representative images of in situ hybridization assays with a *Dbx2* probe performed on (d) coronal sections of adult (5 months) mouse brain cut at the level of the lateral ventricle (LV) or (e) on transversal sections of E11 mouse embryonic spinal cord. *Dbx2* staining is detectable in the SVZ region adjacent to the LV (arrows in d) of the adult brain and in the intermediate embryonic spinal cord region (e). Scale bars, 75 μm (d) and 160 μm (e). (f) qRT‐PCR analysis of *Dbx2* expression in *Tlx‐GFP*‐positive NSPCs freshly cell sorted from adult (7 months) and aged (18 months) mouse brain. Data show mean ± *SEM* following normalization to adult NSPC samples; *n* = 3 biological replicates. *p < .05, Student's *t* test. (g) Analysis of published (Apostolopoulou et al., 2017) RNA‐seq data shows that *Dbx2* levels increase with SVZ age. Data show mean ± *SEM*;* n* = 3 biological replicates. *p < .05, **p < .01, Student's *t* test. mo, months old

This strategy led us to focus on *Dbx2*, which encodes for a homeodomain‐containing transcription factor (Shoji et al., 1996). *Dbx2* is implicated in spinal cord development (Pierani, Brenner‐Morton, Chiang & Jessell, 1999), but has not been previously associated with adult neurogenesis or NSPC aging. *Dbx2* expression was significantly increased in aged NSPCs compared with young adult samples (*p*_adj_ = .04; limma moderated *t* test). Corresponding epigenetic changes were present near to the *Dbx2* promoter, including increased H3K4me3 signal and reduced DNA methylation at an overlapping CGI (Figure 5a). *Dbx2* levels remained higher in aged neurospheres than in young adult cultures after prolonged passaging in vitro (Figure 5b). Furthermore, *Dbx2* was upregulated in adult NSPC cultures upon switching from proliferating to differentiating conditions (Figure 5c), suggesting that elevated *Dbx2* expression is associated with reduced NSPC proliferation.

We next examined *Dbx2* expression in SVZ NSPCs in vivo. In situ hybridization assays on sections of mouse telencephalon suggested that *Dbx2* is transcribed in adult SVZ (Figure 5d), in addition to its previously described expression domain in the embryonic spinal cord (Figure 5e; Shoji et al., 1996). To quantitatively assess *Dbx2* levels, we used qRT‐PCR to compare *Tlx‐GFP*‐positive SVZ NSPCs freshly isolated from adult (7 months) and aged (18 months) mice (Feng et al., 2013). The results showed that *Dbx2* levels were higher in the aged sorted NSPCs compared with adult, with a similar fold change to that detected in aged SVZ‐derived NSPC cultures (Figure 5f). Finally, by re‐analysing a recently published RNA‐seq data set of SVZ tissues dissected at different ages, we detected a significant increase in *Dbx2* levels between 2‐months and 18‐months SVZ samples (Figure 5g) (Apostolopoulou et al., 2017). These results show that *Dbx2* is upregulated in aged SVZ NSPCs in vitro and in vivo, and raise the possibility that *Dbx2* has a functional role in driving age‐associated changes in NSPC proliferation.

### 2.4 *Dbx2* overexpression impairs neurosphere growth in young adult SVZ NSPCs

Aged SVZ NSPCs form smaller neurospheres and adherent colonies compared with young adult NSPCs, and this correlates with their decreased proliferation capacity in vitro (Ahlenius et al., 2009; Corenblum et al., 2016; Daynac et al., 2014, 2016; L'Episcopo et al., 2013; Zhu et al., 2014). We also observed a proliferation defect in NSPCs derived from the aged mice (Figures S1B,C).

**Figure 6:**
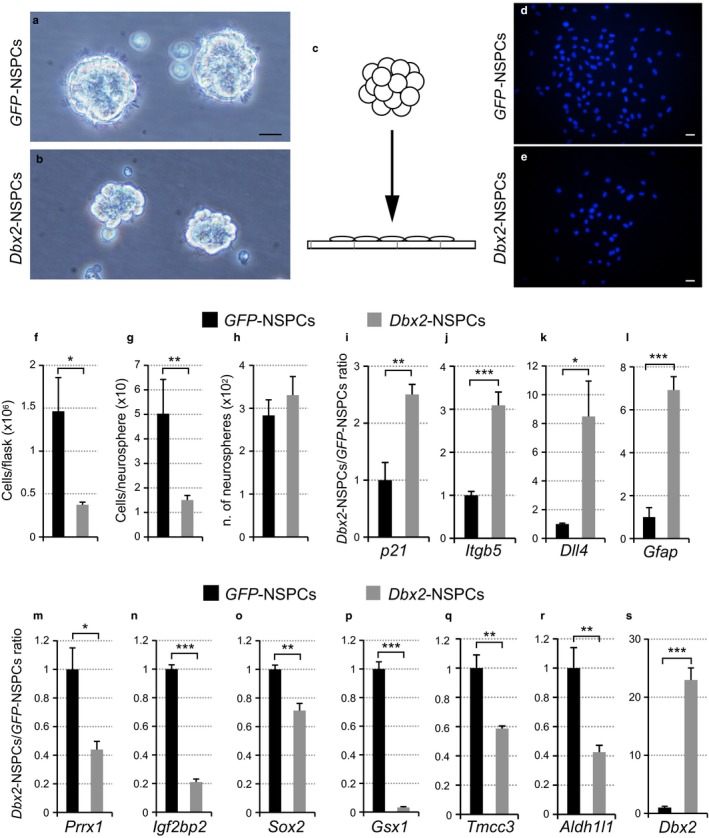
Elevated *Dbx2* expression in young adult SVZ NSPCs impairs neurosphere growth and alters the expression of genes involved in NSPC self‐renewal and differentiation. (a–b) Neurospheres from *Dbx2*‐NSPCs are smaller in comparison with *GFP*‐expressing aggregates. Scale bar, 20 μm. (c) Schematic of the procedure used for quantification of neurosphere size. Neurospheres were allowed to attach on glass coverslips for approximately six hours, followed by nuclear staining and quantification of the number of nuclei contained in each neurosphere‐derived adherent colony. (d, e) Hoechst nuclear staining of representative colonies generated by nonadherent culture of transgenic NSPCs, followed by attachment of neurospheres to glass coverslips. Scale bar, 20 μm. (f–h) Quantification of the average number of cells per 25 cm^2^ flask (f), of the average number of cells per neurosphere (g) and of the average number of neurospheres (h) found in nonadherent cultures of transgenic NSPCs. Data show mean ± *SEM*;* n* = 4 (f) and *n* = 6 (g,h) biological replicates. *p < .05; **p < .01, Mann–Whitney test. (i–s) qRT‐PCR analysis of gene expression in nonadherent cultures of *Dbx2*‐NSPCs. (i–l), upregulated genes; (m–r), downregulated genes; (s) confirmation of increased *Dbx2*‐expression in *Dbx2*‐NSPC cultures. Data show mean ± *SEM* following normalization to *GFP*‐NSPC samples; *n* = 4 biological replicates. *p < .05; **p < .01; ***p < .001, Student's *t* test

To address whether elevated levels of *Dbx2* could recapitulate this proliferation phenotype, we generated young adult NSPC lines that constitutively expressed either a *Dbx2* transgene (*Dbx2*‐NSPCs) or a *GFP* control transgene (*GFP*‐NSPCs). Immunofluorescence microscopy using an anti‐Nestin antibody confirmed the homogeneous NSPC identity of the cultures when maintained in adherent or nonadherent proliferating culture conditions (Figure S2A–H). Furthermore, an anti‐Dbx2 antibody detected increased Dbx2 protein in *Dbx2*‐NSPCs compared to *GFP*‐NSPCs (Figure S2I–P). Quantification of nonviable (trypan blue‐positive) cells and proliferating (Ki67‐positive) cells showed a slight increase in cell death and a slight decrease in the proportion of cycling cells in *Dbx2*‐NSPC cultures (Figure S2Q,R), suggesting that elevated *Dbx2* levels may reduce the self‐renewing capacity of NSPCs. To explore this further, we cultured *GFP*‐NSPCs and *Dbx2*‐NSPCs in nonadherent conditions and, after 4–7 days, quantitated the number and the size of the resulting neurospheres. *Dbx2‐*NSPCs produced smaller neurospheres compared with the *GFP*‐NSPCs (Figure 6a–e). The mean cell number of individual neurospheres in *Dbx2*‐NSPC cultures was approximately 30%–40% that of *GFP*‐NSPCs, with a similar decrease in the mean total cell count (Figure 6f,g). The average number of neurospheres formed did not differ between the *GFP*‐NSPC and *Dbx2*‐NSPC cultures (Figure 6h). These proliferation defects were confirmed with an independent pair of *GFP*‐NSPC and *Dbx2*‐NSPC lines (Figure S3A–E).

### 2.5 *Dbx2* modulates the expression of age‐associated regulators of NSPC proliferation and differentiation

We investigated the *Dbx2*‐mediated transcriptional programme in SVZ NSPCs by focusing on a cohort of genes that are transcriptionally altered upon aging in NSPCs (our data) and SVZ tissue (Apostolopoulou et al., 2017) and are associated with NSPC proliferation and differentiation. *p21*, a key cell cycle inhibitor and age‐associated negative regulator of NSPC proliferation (Akizu et al., 2016), was upregulated in *Dbx2*‐NSPCs (Figure 6i) as were *Itgb5*,* Dll4* and *Gfap* (Figure 6j–l). Conversely, *Prrx1*,* Igf2bp2* and *Sox2* were downregulated in *Dbx2*‐NSPCs (Figure 6l–n), which is a change that is consistent with the positive influence of these genes on NSPC proliferation (Fujii, Kishi & Gotoh, 2013; Nishino, Kim, Zhu, Zhu & Morrison, 2013; Shimozaki, Clemenson & Gage, 2013). Additional genes that were expressed at lower levels in *Dbx2*‐NSPCs included *Gsx1*,* Tmcc3* and *Aldh1 l1* (Figure 6p–s).

Many of these transcriptional responses were mirrored by corresponding changes in aged NSPCs and SVZ tissue. For example, *Prrx1* and *Igfbp2* were downregulated in aged NSPCs and in aged SVZ, similar to *Dbx2*‐NSPCs (Figure 1b, Figure S4A–D, Table S1). *Dll4* was less expressed, and *Tmcc3* more expressed, in aged (18 months) NSPCs (Figure S4E,F), recapitulating the changes observed in 18‐months SVZ (Figure S4G,H). Of note, even older SVZ samples (22 months) showed a significant increase of *Dll4* transcripts and a reduction in *Tmcc3* expression (Figure S4G,H), and this pattern matches the changes detected in *Dbx2*‐NSPCs (Figure 6k,q). Finally, *Gsx1* and *Itgb5* showed transcriptional differences between young adult and aged SVZ (Figure S4K–L) that were similar to those observed in Dbx2‐NSPCs (Figure 6p,j), even though the expression changes did not match the aged NSPCs (Figure S4I,J).

We confirmed these transcriptional changes in four ways. First, the above described gene expression differences were reproducible in an independent pair of *Dbx2*‐NSPC and *GFP*‐NSPC lines (Figure S3F–P). Second, similar effects were detected in adherent proliferating or differentiating NSPC cultures (Figure S5), indicating that the changes were not due to differences in the size or composition of *Dbx2* and *GFP* neurospheres. Third, NSPCs with doxycycline‐controlled expression of *Dbx2* phenocopied the proliferation defects (Figure S6A–D) and the gene expression changes (Figure S6E–N) that were detected in NSPCs that constitutively overexpressed *Dbx2*. Finally, the opposite gene expression changes were observed for a subset of genes (*Itgb5*,* Dll4*,* Gsx1*,* Aldh1l1*) following a partial (35%) reduction in *Dbx2* mRNA by means of a shRNA plasmid (Figure S7).

These results lead us to propose that altered levels of *Dbx2* can affect adult NSPC self‐renewal and function by inducing a specific transcriptional programme that includes age‐associated regulators of NSPC proliferation and differentiation.

## 3 DISCUSSION

Mouse SVZ and SGZ are complex niches where neurogenesis persists throughout adult life due to a finely regulated balance among NSPC self‐renewal, expansion and differentiation. During aging, this regulation is progressively altered, and neurogenic output is curtailed. To investigate the intrinsic molecular changes upon NSPC aging, we report here a comprehensive set of transcriptional and epigenetic maps in young adult and aged SVZ NSPCs. We found that the NSPC epigenome was largely unchanged upon aging, which is broadly consistent with prior studies that profiled somatic stem cells from other aged tissues (Beerman et al., 2013; Bocker et al., 2011; Bork et al., 2010; Fernandez et al., 2015; Liu et al., 2013; Sun et al., 2014). In contrast to previous reports, we did not detect an age‐associated broadening of H3K4me3 (as in blood stem cells; Sun et al., 2014) and H3K27me3 (as in muscle satellite stem cells; Liu et al., 2013) domains in NSPCs, nor a global acquisition in DNA methylation levels (as in blood stem cells; Beerman et al., 2013; Sun et al., 2014). It is currently unclear what impact these epigenetic changes might have on the aged stem cells, and whether the differences are due to the varied stem cell properties from each tissue, such as cellular potential or the turnover rate, or perhaps influenced by external signals from their niches.

Despite the overall similarities, we did identify several hundred genes with significant differences in the levels of transcription and/or epigenetic modifications in NSPCs from the aged SVZ. These data will serve as an important resource for normal and aging studies and could be used to identify biomarkers and candidate regulators of NSPC aging. As an initial step, we identified and characterized the homeobox gene *Dbx2* as a putative age‐associated regulator of NSPC functional decline. Increased *Dbx2* expression in young adult NSPCs promoted age‐related phenotypes, including the reduced proliferation of NSPC cultures and the altered expression levels of age‐associated regulators of NSPC proliferation and differentiation. In particular, elevated *Dbx2* expression decreased the size, but not the number, of neurospheres generated from adult SVZ NSPCs, supporting a causal link between *Dbx2* upregulation and the defective proliferation of aged NSPCs. *Dbx2* overexpression slightly increased the fraction of nonviable cells and decreased the fraction of cycling cells, suggesting that cell death and cell cycle exit contribute to the reduced expansion of the transgenic neurospheres. Nonetheless, as the majority of the cells remained positive for proliferation markers, alternative changes, including lengthening of the cell cycle, could be a main driver of the growth phenotype elicited by *Dbx2*. This hypothesis is consistent with the previously described alterations in the cell cycle of aged NSPCs (Apostolopoulou et al., 2017; Daynac et al., 2014, 2016; Stoll et al., 2011). *Dbx2* is part of a cohort of transcription factor genes that are enriched in quiescent NSPCs of the SGZ and SVZ and are downregulated in NSPCs actively engaged in cell proliferation and neurogenesis (Codega et al., 2014; Shin et al., 2015), thus suggesting that *Dbx2* may negatively regulate NSPC proliferation in both adult neurogenic niches. Elevated *Dbx2* expression in adult NSPCs also phenocopied several of the molecular effects of aging, including the altered expression of key genes such as *Sox2* and *p21* that are associated with proliferation and differentiation. Thus, *Dbx2* may act as a crucial regulator of age‐dependent transcriptional programmes, as also suggested by the reversed trend of some molecular markers of SVZ aging following moderate *Dbx2* knockdown in aged NSPCs. As cell proliferation was not affected by this partial knockdown, an exciting future line of research will be to abrogate *Dbx2* function in aged NSPCs by knockout approaches in vitro and in vivo. Furthermore, investigating the direct targets of Dbx2 might help to expand the gene regulatory networks involved in NSPC dysfunction.

*Prrx1* and *Igf2bp2* are additional candidate regulators of NSPC functional decline during SVZ aging. Both genes were downregulated in NSPCs from the aged SVZ, and for *Igf2bp2* this correlated with increased levels of repressive epigenetic marks at the promoter. *Prrx1* is expressed in NSPCs of the SVZ and the SGZ and promotes proliferation of SGZ NSPCs at the expense of neuronal differentiation (Shimozaki et al., 2013). *Igf2bp2* is expressed in embryonic cortical progenitors where it promotes neurogenesis at the expense of astrogenesis (Fujii et al., 2013), but its role in the postnatal SVZ has not been investigated. *Igf2bp1* supports proliferation and prevents premature differentiation of embryonic cortical progenitors, but its expression is extinguished postnatally (Nishino et al., 2008). It is tempting to speculate that the role of *Igf2bp1* in maintaining embryonic NSPC proliferation is superseded by *Igf2bp2* in the postnatal SVZ, and that the combined decay of *Igf2bp1/2* levels underlies the progressive decline of NSPC proliferation during postnatal life. Increased *Dbx2* expression in adult NSPCs caused the downregulation of *Prrx1* and *Igf2bp2*, providing a potential explanation, along with the transcriptional effects on *Sox2* and *p21*, for the antiproliferative activity of *Dbx2*.

In conclusion, by comparing the molecular profiles of young adult and aged SVZ, our work suggests that aging does not cause widespread gene dysregulation in NSPCs, but acts through the modulation of specific gene expression programmes. Among them, we have identified a transcriptional pathway involving the upregulation of the homeodomain protein Dbx2 in aged NSPCs. Dbx2, in turn, can influence a cohort of age‐associated regulators of NSPC function, and restrain NSPC proliferation. These results provide a platform to investigate how changes in extrinsic signals acting in the SVZ niche due to aging, injury or disease could impinge on intrinsic transcriptional mechanisms controlling adult neurogenesis.

## 4 EXPERIMENTAL PROCEDURES

### 4.1 Mouse NSPC isolation, culture and manipulation

Young adult and aged SVZ NSPCs were derived from 3 months or 18 months male C57BL/6 J/Babr mice, respectively. Methods for their derivation and subsequent in vitro culture were reported previously (Soldati et al., 2015) and are described in the Supplemental Experimental Procedures. We combined SVZ tissues from five mice to form one NSPC derivation, and we repeated this procedure three times on different days to obtain three independent replicates. The neurospheres used for the transcriptomic and epigenomic assays were harvested 5–6 days after seeding SVZ NSPCs at the second passage postdissection. Summing the first and second passages, SVZ NSPCs were cultured for a total of 13–20 days postdissection before being processed for molecular analyses. Experimental procedures for NSPC derivation were performed in accordance with EU Directive 2010/63/EU and were approved by the Ethical Committee for Animal Research of the Italian Ministry of Health according to national regulations (Art. 7, D.Lgs. n. 116/1992). Detailed methods for the transcriptional and epigenetic profiling are provided in the Supplemental Experimental Procedures.

## ACCESSION NUMBERS

Sequencing data are available at GEO under accession number GSE101610.

## CONFLICT OF INTEREST

None declared.

## AUTHOR CONTRIBUTIONS

G.L. performed conceptualization, resources, funding acquisition, investigation, methodology, supervision, validation, visualization and writing. P.S.N. performed investigation. P.E. and M.A.K. were involved in investigation and visualization. Y.‐L.P. and C.L.N. performed investigation and methodology. B.S. carried out methodology and formal analysis. S.B. performed conceptualization and funding acquisition. H‐K.L. involved in investigation and supervision. P.B. was involved in visualization, resources and funding acquisition. E.C. performed conceptualization, resources, funding acquisition, investigation, methodology, visualization and writing. P.J.R.‐G. performed conceptualization, resources, formal analysis, funding acquisition, supervision, validation, visualization and writing.
