# Supplementary material for: Molecular profiling of aged neural progenitors identifies Dbx2 as a candidate regulator of age‐associated neurogenic decline
Source: Aging Cell. 2018 Mar 5;17(3):e12745. doi: 10.1111/acel.12745 (PMC5946077; doi:10.1111/acel.12745)
Supplement: Supplementary file 1 [file ACEL-17-e12745-s001.pdf]

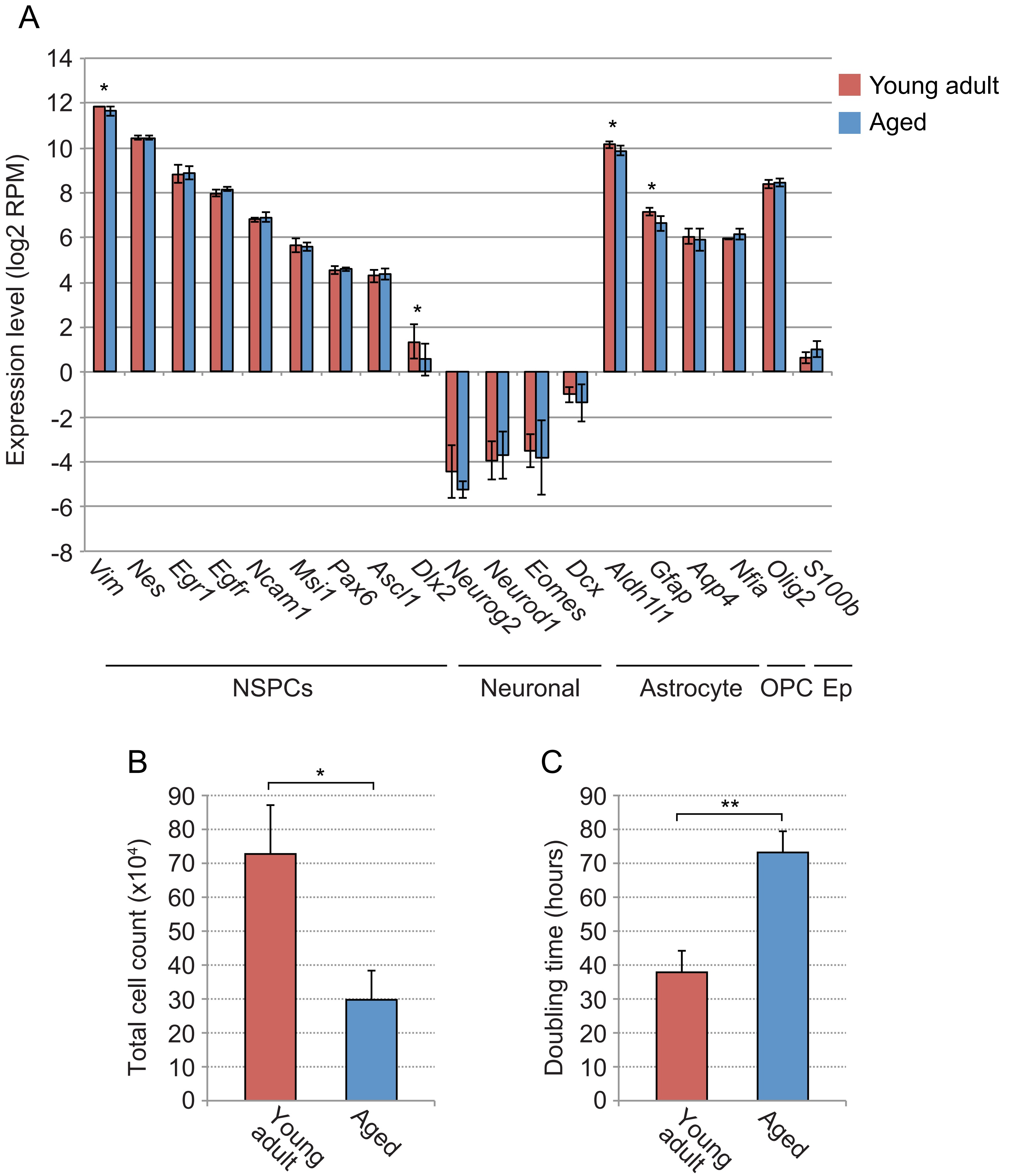

Fig. S1. Proliferation defects of aged NSPCs are indepdendent of neurosphere composition.
(A) RNA-seq analysis of well-established marker genes of NSPCs, early neuronal differentiation, astrocytes, oligodendrocyte progenitor cells (OPC), and ependyma (Ep) in young adult and aged NSPCs. Data show mean ± s.d.; n=3 biological replicates. *, FDR<0.05, Limma-moderated t-test with multiple testing correction.
(B–C) Cell proliferation defects in aged NSPCs. **(B)** Reduction of cell number in aged neurosphere cultures after one passage. Data show mean ± s.e.m.; n=4 biological replicates. *, p<0.05, Student's t-test. **(C)** Cell doubling time is extended in aged NSPCs, as measured between passages 5 and 6. Data show mean ± s.e.m.; n=4 biological replicates. **, p<0.01, Student's t-test.

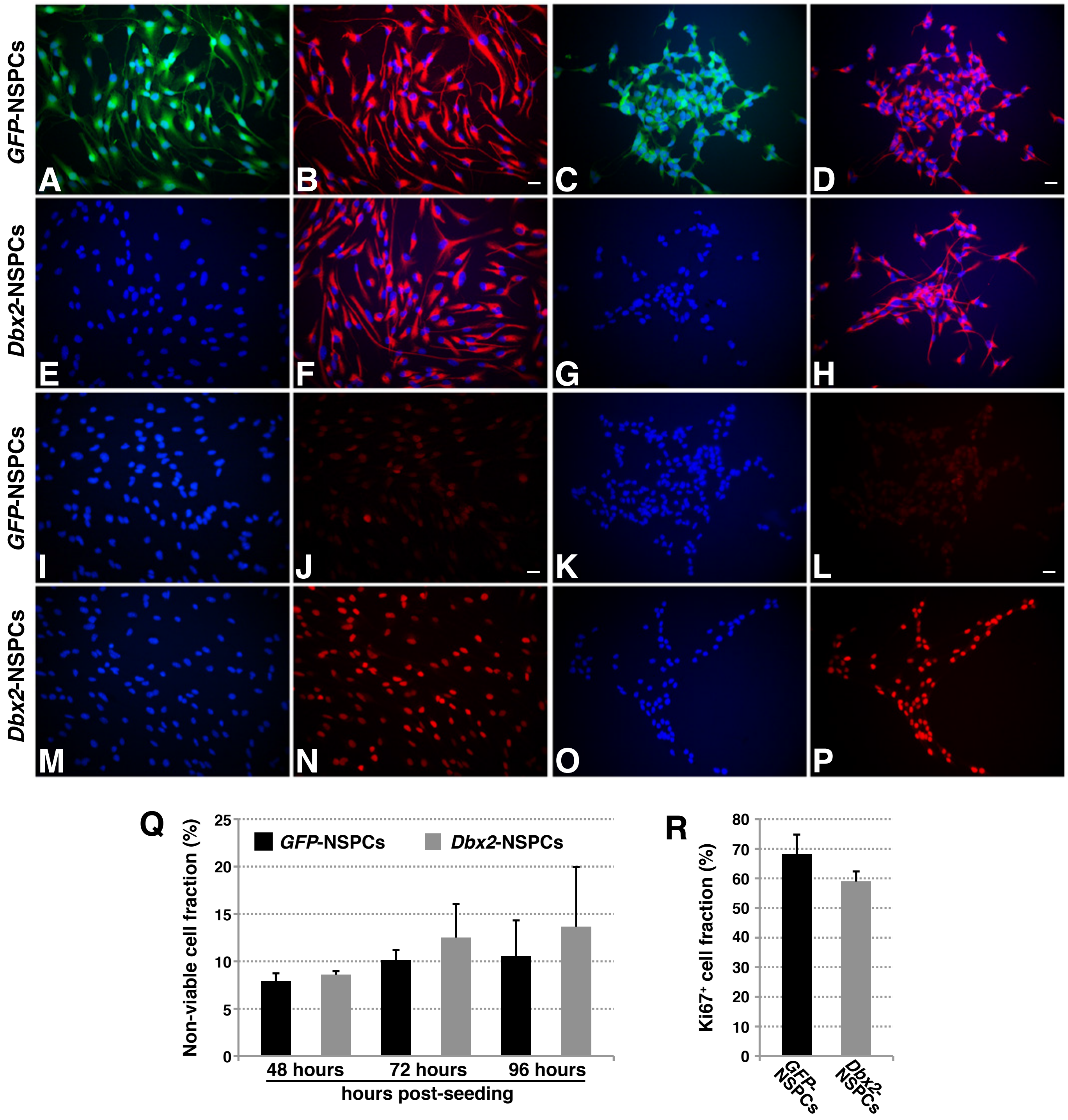

Fig. S2. *GFP*-NSPCs and *Dbx2*-NSPCs are uniformly Nestin-positive, and show altered viability and proliferation.

(A–H) Immunofluorescence microscopy with an anti-Nestin antibody on *GFP*-NSPCs (A–D) and *Dbx2*-NSPCs (E–H) shows that ~100% of cells in both cultures are positive for the neural progenitor marker Nestin (red). Images taken from adherent NSPCs (A,B,E,F) and neurospheres that were attached to glass coverlsips (C,D,G,H), following culture in proliferating conditions. GFP signal (green) and Hoechst nuclear staining (blue) are shown. Scale bar, 20 µm.

(I–P) Immunofluorescence microscopy with an anti-Dbx2 antibody (red) on adherent NSPCs (I,J,M,N) and attached neurospheres (K,L,O,P), following culture in proliferating conditions. Dbx2 protein levels are higher in Dbx2-NSPCs in comparison with GFP-NSPCs. Hoechst nuclear staining (blue) is shown. Scale bar, 20 µm.

(Q) Quantification of the percentage of non-viable cells by trypan blue staining in adherent transgenic NSPCs at the indicated times after cell seeding reveals a slight increase in cell death in *Dbx2*-NSPCs. Data show mean ± s.e.m; n=3 biological replicates for each time point.

(R) Quantification of the percentage of Ki67-positive cells in attached neurospheres using immunofluorescent microscopy reveals a slight decrease in the proportion of cycling cells in *Dbx2*-NSPCs. Data show mean ± s.e.m.; n=3 biological replicates. Over 2500 cells were scored for each replicate sample.

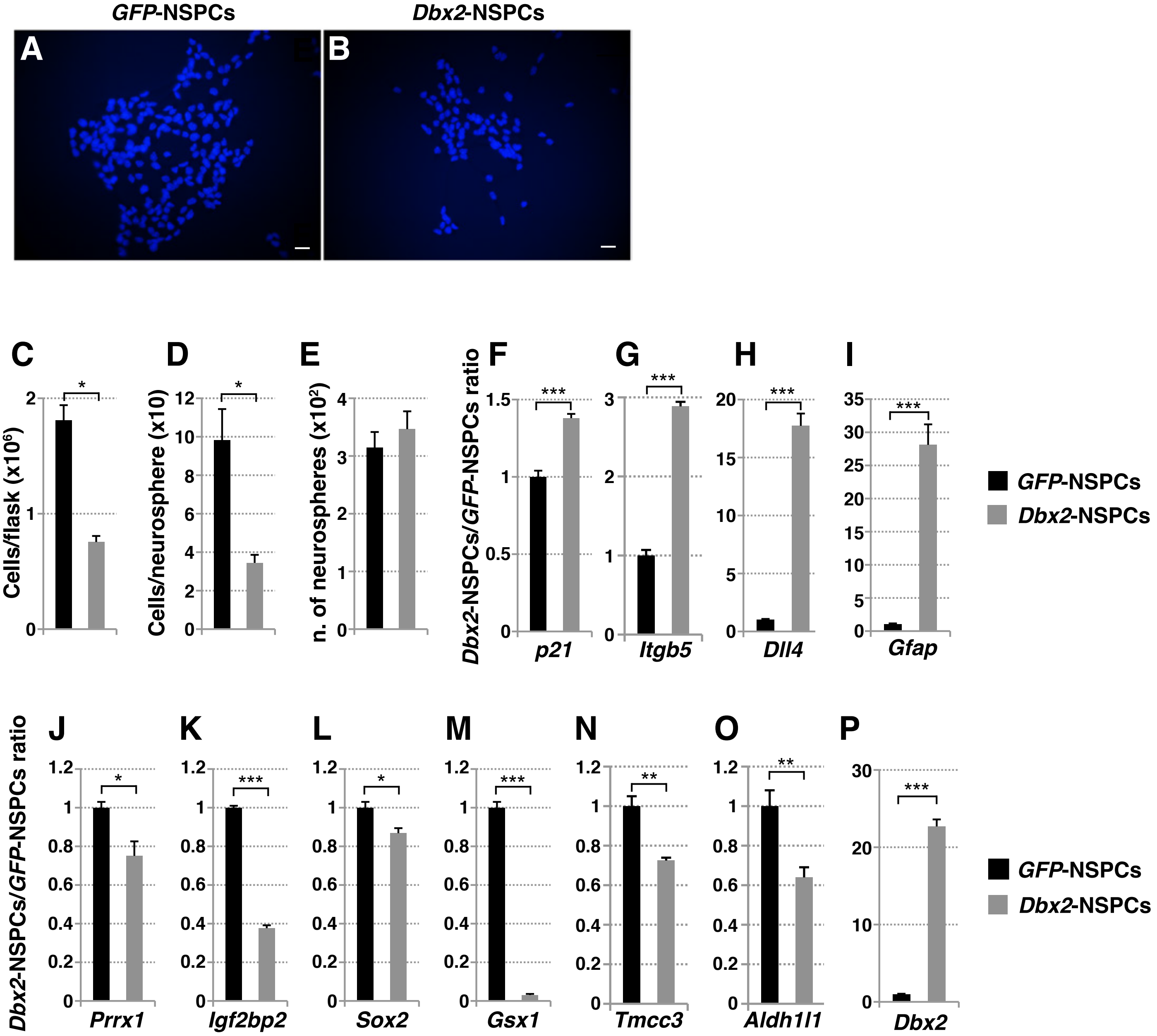

Fig. S3. Validation of the effects of *Dbx2* overexpression in an independent pair of *GFP*-NSPCs and *Dbx2*-NSPCs.

(A-B) Hoechst staining of colonies generated by non-adherent culture of an independent pair of transgenic NSPCs, followed by attachment of neurospheres to glass coverslips. Confirming data in Fig. 6D,E with a different set of *GFP*-NSPCs and *Dbx2*-NSPCs, the number of cells in the *Dbx2*-overexpressing sample is less than half the number present in the *GFP*-expressing sample. Scale bar, 20 μm.

(C-E) Quantification of the average number of cells per 25 cm^2 flask **(C)**, of the average number of cells per neurosphere **(D)** and of the average number of neurospheres **(E)** found in non-adherent cultures of an independent pair of *GFP*-NSPCs and *Dbx2*-NSPCs. Confirming data shown in Fig. 6F-6H, *Dbx2* overexpression causes a significant reduction in neurosphere size and in the total cell count in *Dbx2*-NSPC cultures in comparison with *GFP*-NSPC cultures, whereas the number of neurospheres is similar. Data show mean ± s.e.m.; n=4 biological replicates. *, $p<0.05$, Mann-Whitney test.

(F-P) qRT-PCR analysis of gene expression in non-adherent cultures of an independent pair of *GFP*-NSPCs and *Dbx2*-NSPCs confirms data shown in Fig. 6I-6S. Data show mean ± s.e.m.; n=4 biological replicates. *, $p<0.05$; **, $p<0.01$; ***, $p<0.001$, Student's t-test.

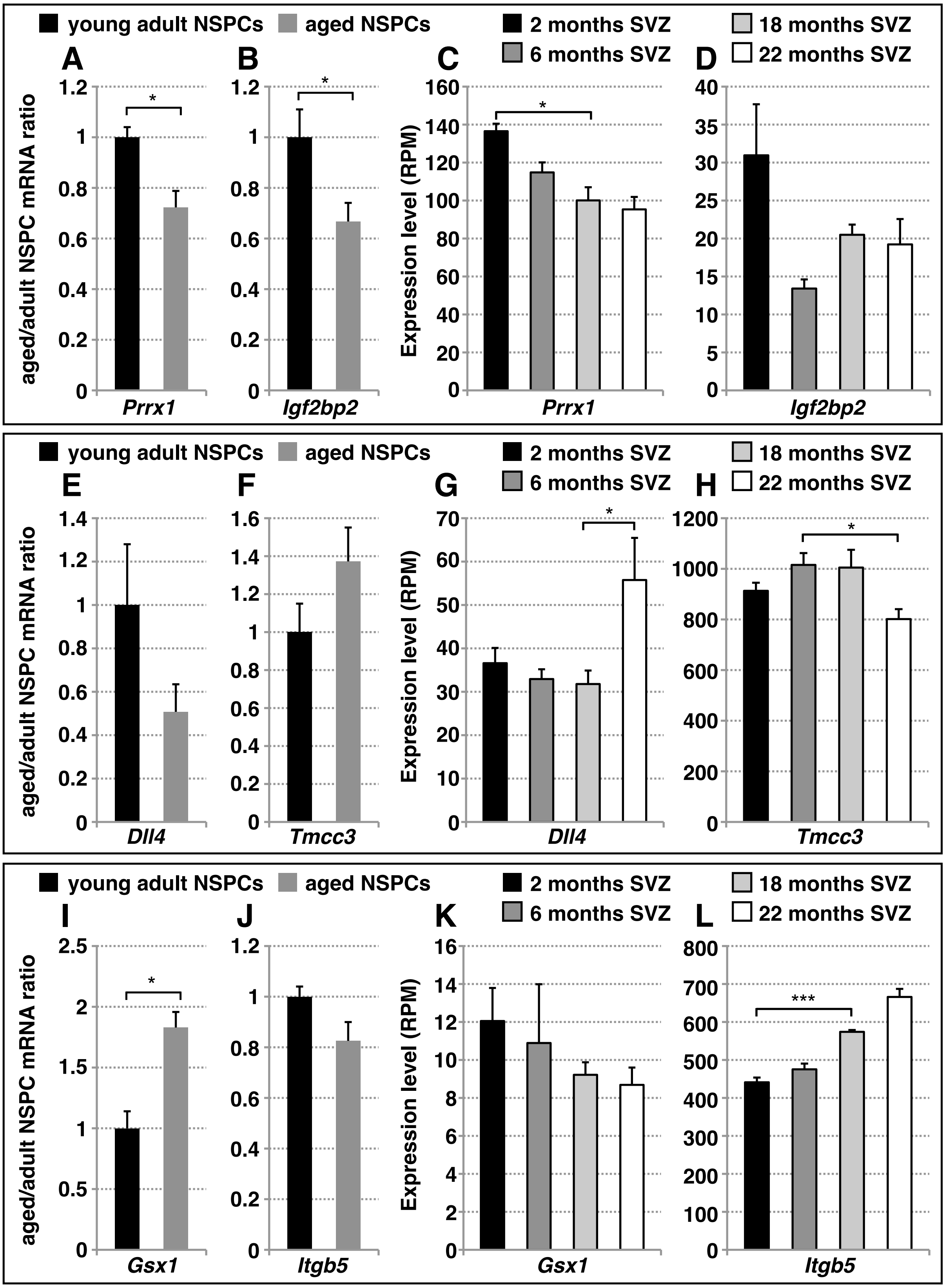

Fig. S4. Identification of candidate genes implicated in self-renewal and/or differentiation of SVZ NSPCs during ageing.

(A,B; E,F; I,J) qRT-PCR analysis of expression of six genes in non-adherent cultures of young adult or aged NSPCs. Data show mean ± s.e.m. following normalization to adult NSPC samples; n=4 biological replicates. *, p<0.05, Student's t-test.

(C,D; G,H; K,L) RNA-seq data of SVZ dissected from mice at 2mo, 6mo, 18mo and 22mo (Apostolopoulu et al. 2017), for the same six genes. Data show mean ± s.e.m.; n=3 biological replicates. *, p≤0.05; **, p<0.01; ***, p<0.001 Student's t-test.

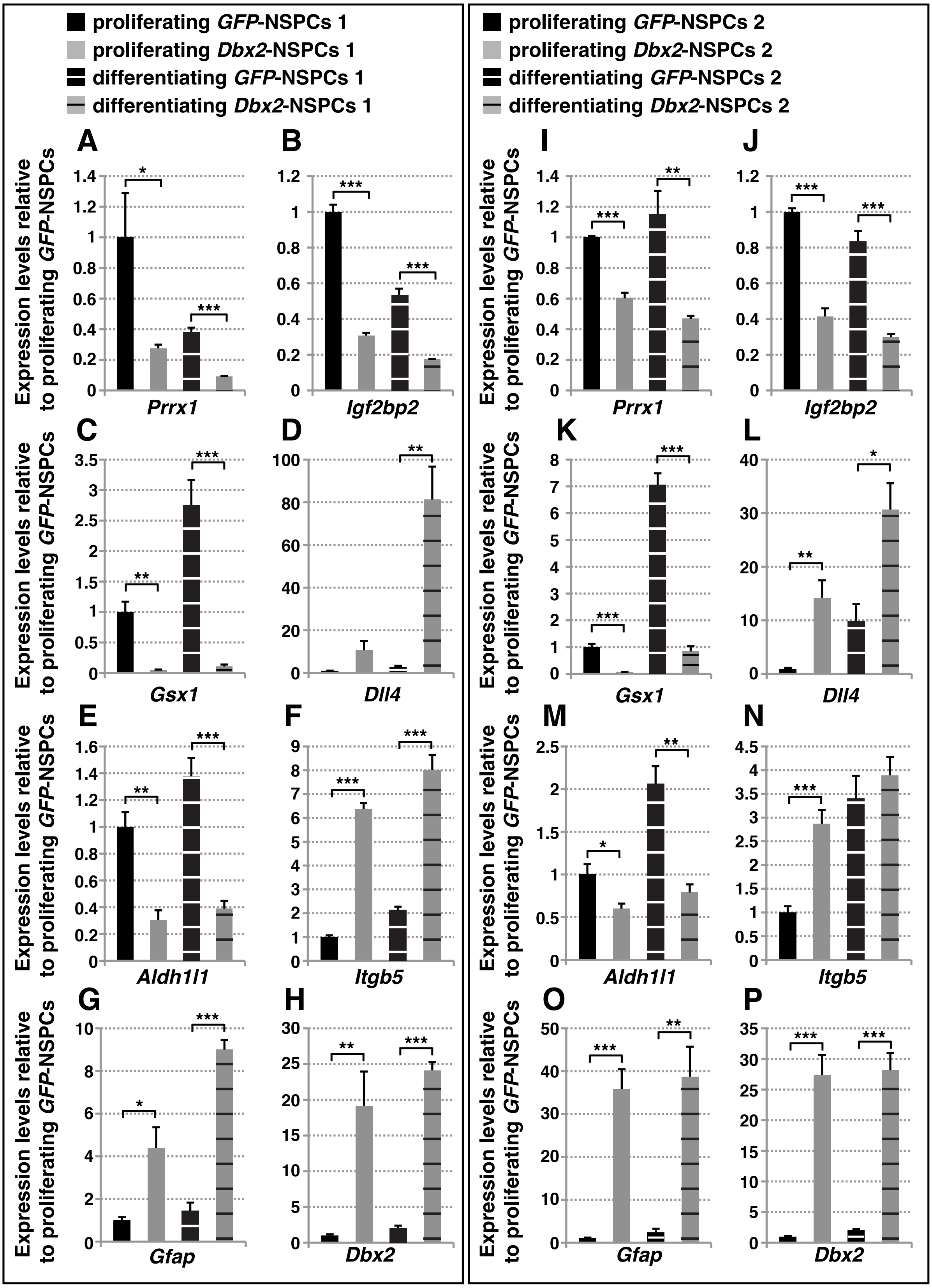

Fig. S5. Gene expression changes caused by *Dbx2* overexpression in non-adherent cultures are also detectable in adherent cultures of young adult SVZ NSPCs.

(A-P) qRT-PCR analysis of gene expression in adherent cultures of *GFP*-NSPCs and *Dbx2*-NSPCs that were maintained in proliferating conditions or cultured for three days in differentiation conditions devoid of EGF, as indicated. **(A-H)** and **(I-P)** report the results obtained with two different pairs of transgenic NSPCs generated from independent derivations of adult SVZ NSPCs, respectively. Data show mean ± s.e.m. following normalization to proliferating *GFP*-NSPC samples; n=4 biological replicates. *, p≤0.05; **, p<0.01; ***, p<0.001, Student's t-test.

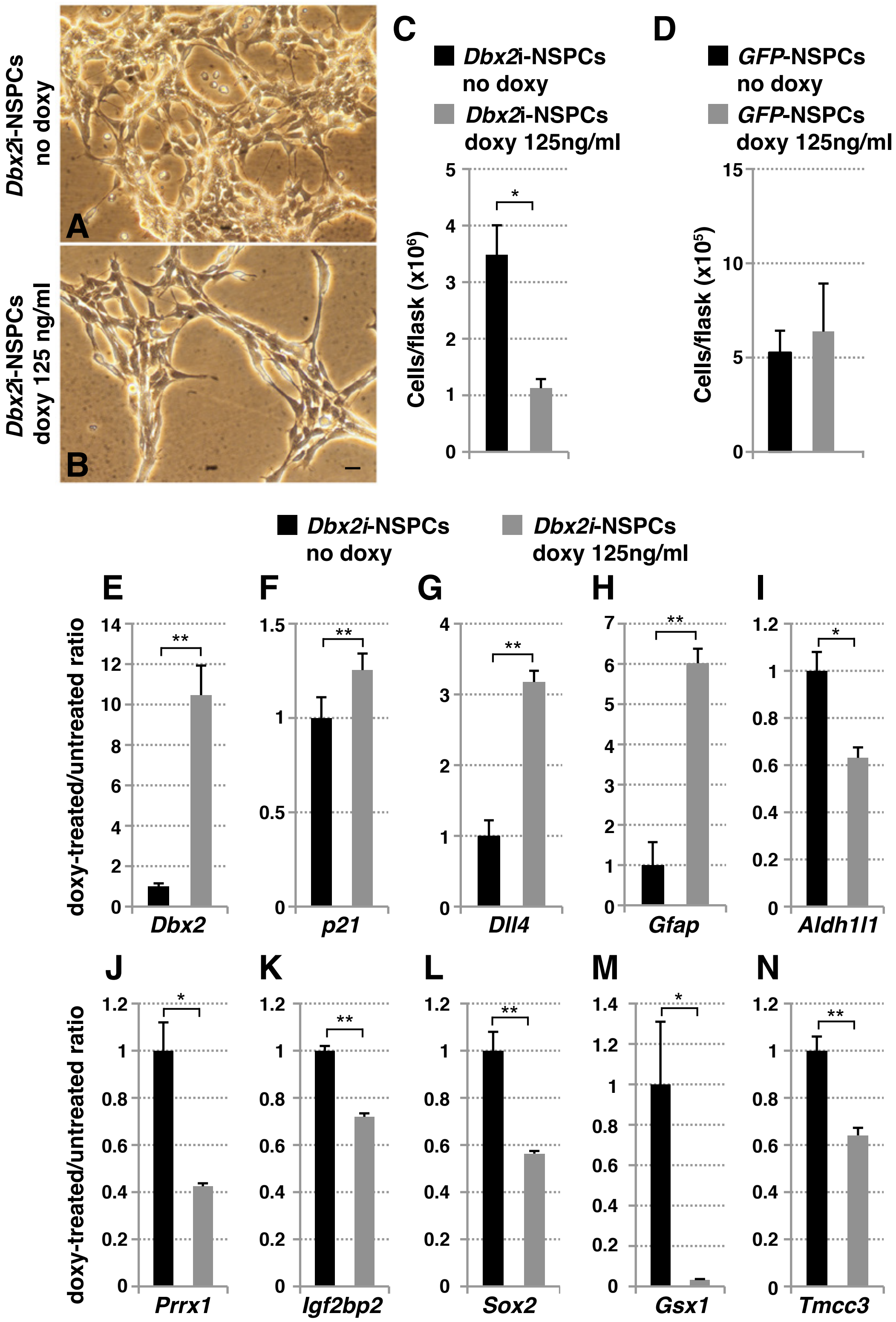

Fig. S6. Inducible *Dbx2* overexpression recapitulates the constitutive *Dbx2*-NSPC proliferation and transcriptional phenotypes.

(A-B) Phase microscopy images of young adult NSPCs stably containing a doxycycline (doxy) inducible *Dbx2* plasmid (*Dbx2*i-NPSCs), treated with either **(A)** 0 or **(B)** 125ng/ml doxy during culture in proliferating conditions.

(C) Reduction in cell number upon *Dbx2* induction. Quantification of the average number of *Dbx2*i-NPSCs per 25 cm^2 flask with and without doxy. Data show mean ± s.e.m.; n=4 biological replicates. *, $p<0.05$, Mann-Whitney test.

(D) Treatment of control cells, *GFP*-NPSCs, with doxy had no effect on cell number. Data show mean ± s.e.m.; n=2 biological replicates.

(**E-N**) qRT-PCR analysis of gene expression in adherent cultures of *Dbx2*i-NPSCs with and without doxy. Data show mean ± s.e.m. following normalization to ‘no doxy’ samples; n=4 biological replicates.
*, $p\leq0.05$; **, $p<0.01$, Student's t-test.

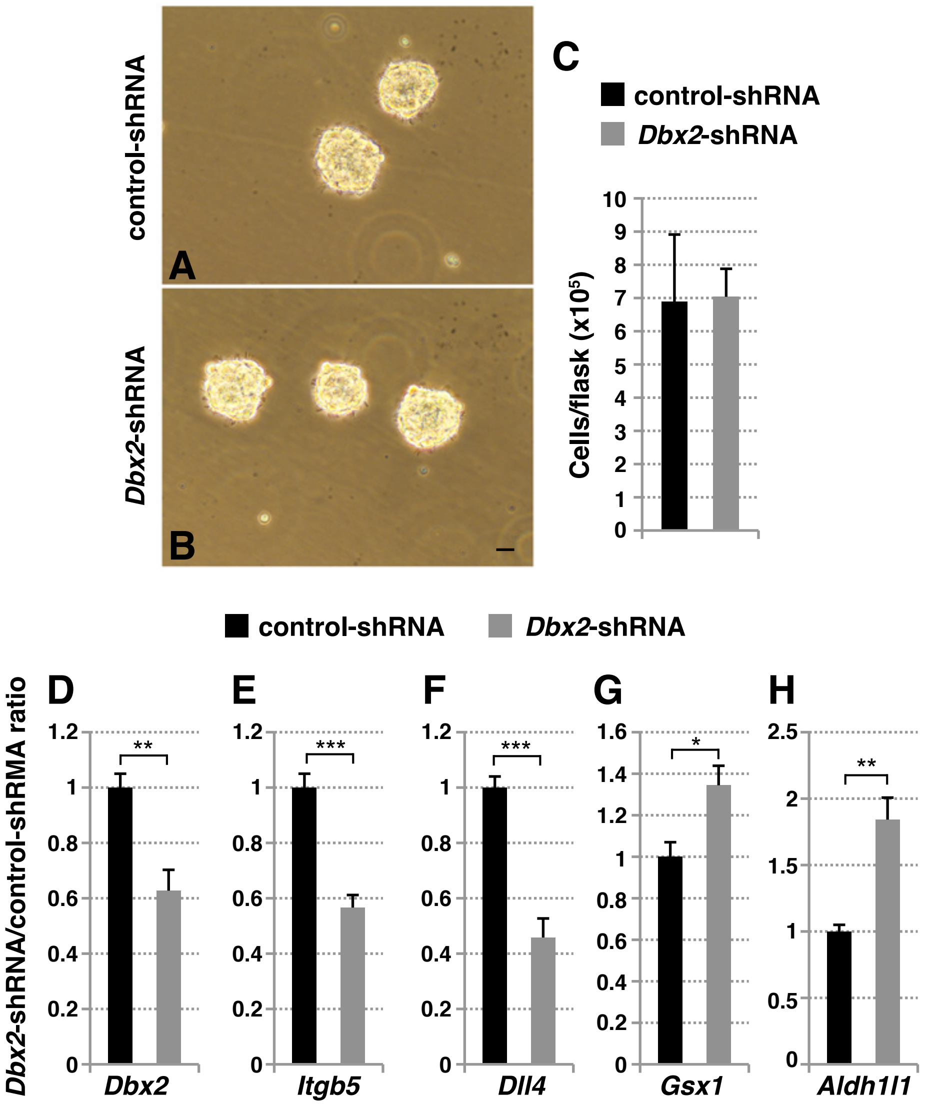

Fig. S7. Partial *Dbx2* knockdown causes opposite transcriptional changes to those elicited by *Dbx2* overexpression.

(A-B) Phase microscopy images of adult NSPCs neurospheres stably containing an shRNA plasmid targeting either **(A)** a non-silencing control or **(B)** *Dbx2*.

(C) No reduction in cell number upon modest *Dbx2* knockdown. Quantification of the average number of cells per 25 cm^2 flask for control shRNA and *Dbx2* shRNA expressing NSPCs that were cultured in non-adherent proliferating conditions. Data show mean ± s.e.m.; n=4 biological replicates.

(D-H) qRT-PCR analysis of gene expression in non-adherent cultures of NSPCs expressing control shRNA or *Dbx2* shRNA. Data show mean ± s.e.m. following normalization to 'control shRNA' samples; n=4 biological replicates. *, p<0.05; **, p<0.01; ***, p<0.001, Student's t-test.

SUPPLEMENTAL TABLES

Table S1. List of differentially expressed genes.

Table S2. List of differentially methylated regions.

Table S3. List of regions with differential promoter H3K4me3 levels.

Table S4. List of regions with differential promoter H3K27me3 levels.

Table S5. List of candidate genes implicated in SVZ NSPC aging.

SUPPLEMENTAL EXPERIMENTAL PROCEDURES

Mouse NSPC culture and manipulation

For neurosphere culture, NSPCs were propagated on uncoated T25 flasks (Corning) in chemically-defined media as described (Soldati et al. 2012; De Luca et al. 2013; Soldati et al. 2015), supplemented with 20 ng/ml human recombinant Epidermal Growth Factor (R&D systems), 10 ng/ml human recombinant Fibroblast Growth Factor-basic (Peprotech), 1:50 B27 supplement (Invitrogen) and 2 μg/ml heparin (Sigma-Aldrich). NSPCs grown as neurospheres were passaged approximately once per week by pelleting the cell aggregates, dissociating with Accutase (Sigma-Aldrich) and seeding on new uncoated flasks at a density of 20-50000 cells/ml. For the establishment of adherent NSPC cultures, dissociated NSPC-derived neurospheres were initially plated in 6-well plates coated with 10 μg/ml poly-ornithine (Sigma-Aldrich) and 5 μg/ml laminin (Sigma-Aldrich) at a density of 10-15000 cells/cm^2 using the same media as described above without heparin. Subsequent passages were performed once or twice per week using Accutase to detach NSPCs and seeding the cells in coated T25 flasks at a density of 10-20000/cm^2. For differentiation assays, adherent NSPCs seeded at a density of 20-25000 cells/cm^2 were induced to differentiate by incubating them in media devoid of EGF from the day after seeding.

To generate transgenic adult SVZ NSPCs, 2-4x10^6 cells expanded in adherent conditions were transfected with 2-4 µg of the following plasmids: pTP6-hrGFP control plasmid (Vallier et al. 2004); pTP6-Dbx2 plasmid, containing the open reading frame of mouse Dbx2 cloned into the EcoRI site of the pTP6 vector (Pratt et al. 2000); epB-Puro-TT-Dbx2 plasmid, containing the open reading frame of mouse Dbx2 cloned in the BamHI/NotI sites of the doxycycline-inducible epB-Puro-TT plasmid (Lenzi *et al*. 2016); pLKO.1-puro shRNA control plasmid (target sequence: 5'-CAACAAGATGAAGAGCACCAA-3'; Sigma-Aldrich); TRC2-pLKO-puro *Dbx2* shRNA plasmid (target sequence: 5'-TGCTGACCCAGGACTCAAATT-3'; Sigma-Aldrich). NSPC transfection was carried out using Amaxa mouse NSC Nucleofector kit (Lonza) on an Amaxa Nucleofector II (Lonza). Transfected cells were seeded in two coated T25 flasks and, starting from the second day after transfection, selected for stable plasmid integration by treatment with 1 µg/ml of puromycin (Sigma-Aldrich). Transgenic cells were expanded as described above for up to 25 passages in vitro since the initial derivation of NSPCs from SVZ tissue, by maintaining them in media containing 1 µg/ml of puromycin. Two different pairs of NSPCs transfected with pTP6-hrGFP and pTP6-Dbx2 plasmids were generated for this study, starting from independent derivations of adult SVZ NSPCs. *Dbx2* expression from the epB-Puro-TT-Dbx2 plasmid was induced by treating transgenic NSPCs with 125 ng/ml doxycycline hyclate (Sigma-Aldrich) diluted from a 1 µg/ml stock in PBS. Cells were routinely expanded in the absence of doxycycline, which was added at the time of seeding for experimental assays and maintained in the media until the cells were harvested for endpoint analyses. In each experiment, half of the cultures were left untreated as an internal control.

For neurosphere assays with transgenic NSPCs, adherent transgenic cultures were seeded in uncoated T25 flasks at a density of either 1000 cells/ml or 25000 cells/ml in media for non-adherent culture supplemented with 600 ng/ml of puromycin. Neurospheres arising in low density cultures were plated after 5-7 days post-seeding in 24-well plates containing glass coverslips coated with 20 µg/ml poly-ornithine and 10 µg/ml laminin and left to attach for 4-6 hours. Attached colonies were subsequently fixed with 4% methanol-free formaldehyde (Pierce) in PBS, followed by Hoechst

nuclear staining and quantification of the total number of neurospheres and the number of cells per sphere in each sample. Neurospheres generated in high density cultures were harvested after 4-8 days post-seeding and dissociated with Accutase, followed by the quantification of the total number of cells per flask and freezing of cell pellets for qRT-PCR. Differentiation of transgenic NSPCs was initiated by culturing adherent NSPCs in EGF-free media as described above, in the presence of 600 ng/ml of puromycin.

Isolation of *Tlx-GFP* NSPCs

Tlx-GFP reporter mice were obtained from the GENSAT project (Gong et al., 2003). Mice were housed according to international standard conditions and all animal experiments complied with local (Regierungspräsidium Karlsruhe) and international guidelines for the use of experimental animals. SVZ tissues from three adult (7 month old) and three aged (18 months) C57BL/6 mice were dissected separately in phosphate buffered saline (PBS) and enzymatically dissociated with 0.05% Trypsin/EDTA (Life Technologies) in HBSS containing 2mM glucose at 37°C for 30 min. During incubation, the tissues were repeatedly triturated with a fire polished Pasteur pipette. Enzyme activity was stopped by addition of equal volume of 4% BSA in Earle's Balanced Salt Solution (EBSS, Life Technologies). Cell suspensions were filtered through a 70µm cell strainer and centrifuged at 1200 rpm for 5 min and the pellets were re-suspended in 0.9 M sucrose in 0.5 x HBSS (Life Technologies). After further centrifugation for 20 min at 2000 rpm, the cell pellets were re-suspended in 2 ml of 4 % BSA in EBSS solution and placed on top of 12 ml of 4 % BSA in EBSS solution, centrifuged again for 9 min at 1500 rpm. The resulting cell pellets were suspended in PBS containing Recombinant RNase Inhibitor, 1 unit/µl reaction (Clontech). After incubation with PI (1:1000) for 2 minutes, *Tlx-GFP* positive cells were sorted on a BD FACS Aria.

Immunocytochemistry and *in situ* hybridization

Immunocytochemistry was performed on adherent NSPCs cultured on glass coverslips coated with 10 µg/ml poly-ornithine and 5 µg/ml Laminin or neurospheres attached to glass coverslips as described above. Cells were fixed with 4% formaldehyde in PBS for 20min, followed by washes with PBS, permeabilization with 0.5% Triton X-100 in PBS, incubation with blocking solution (5% goat serum in PBS) and incubation with blocking solution containing primary antibodies (rabbit anti-Dbx2 (Abcam), 1:1000; mouse anti-Nestin (Millipore), 1:1000; rabbit anti-Ki67 (Abcam), 1:200) overnight at 4ºC. Cells were rinsed with PBS and incubated with blocking solution containing Cy3-conjugated (Jackson Immunoresearch, 1:150) or AF647-conjugated (Life Technologies, 1:500) secondary antibodies and Hoechst nuclear staining for 90min at RT, followed by additional washes with PBS and mounting with Dako mounting media. The percentage of proliferating cells was determined by counting the number of Ki-67 positive cells using the Cell Counter plugin in ImageJ, after applying threshold adjustment.

For *in situ* hybridization analysis on the SVZ, three, 5mo male C57BL/6 mice were transcardiacally perfused with PBS followed by 4% paraformaldehyde in phosphate buffer (0.1M, pH 7.3). The brain was dissected and post-fixed for 2h. For analysis in the embryonic spinal cord, E11.5 embryos (C57BL/6) were immersion-fixed in the same fixative for 3h. Animals were used according to institutional (CBMSO, Madrid), Spanish and European guidelines under the approved protocol PROEX 100/15 (RD 53/2013). Tissues were washed in PBS, incubated in a 30% sucrose solution in PBS and embedded and frozen in a 7.5% gelatin, 15% sucrose solution. Cryostat sections were processed as described (Esteve *et al.* 2011), with a mouse-specific digoxigenin-labeled antisense *Dbx2* riboprobe. The sense probe was used as a negative control. *In vitro* transcription of RNA probes was carried out with T7 RNA polymerase from pJET-*Dbx2* sense and pJET-*Dbx2* antisense plasmids containing mouse *Dbx2* open reading frame in opposite orientations. Sections were analysed and photographed with a Leica DM microscope equipped with a DFC500 digital camera.

Quantitative RT-PCR

Total RNA extraction from frozen cell pellets and qRT-PCR were performed as previously described (Carucci et al. 2017). Relative gene expression levels were determined using Eef1a1 and Rpl19 as reference genes. Total RNA from *Tlx-GFP* isolated cells was harvested with an RNeasy Mini Kit (QIAGEN) and reverse transcribed into cDNA using random primers (dN6, Roche).

Primer sequences

Prrx1_Forward	GGACACATTACCCGGATGCT
Prrx1_Reverse	CGGAACTTGGCTCTTCGGTT
Igf2bp2_Forward	GCCAGACGAGAATGAGGAAGT
Igf2bp2_Reverse	CTGCTGCTTCACCTGCTGTA
Sox2_Forward	CATGGCCCAGCACTACCAG
Sox2_Reverse	TTTGCACCCCTCCCAATTCC
Gsx1_Forward	CCGGATCCCAGACAGTTTCA
Gsx1_Reverse	GCGGGAGAGGTACATGTTGG
p21_Forward	GATATCCAGACATTCAGAGCCACA
p21_Reverse	GGGACCGAAGAGACAACGG
Dll4_Forward	CCCCAACTTTACCGGCTCTA
Dll4_Reverse	GTGGGTGCCTGTGAATCCAG
Gfap_Forward	TCAACGTTAAGCTAGCCCTGG
Gfap_Reverse	CGGATCTGGAGGTTGGAGAAAG
Aldh1l1_Forward	ACCTGCGGATCAAGACTGTG
Aldh1l1_Reverse	GGACAGGAGGGTGCTAAGTC
Itgb5_Forward	AGGAGGCTGTGCTTTGCTT
Itgb5_Reverse	AGGATGGTCATGGCATTGGG
Tmcc3_Forward	ACTGGGACCATATCTTGTGTGC
Tmcc3_Reverse	GCACAGCCGTCCATTCAGAA
Dbx2_Forward	CCCGCCATTCTACTCTGCAT
Dbx2_Reverse	GAGTCCTGGGTCAGCAAAGG
Rpl19_Forward	AGACCAAGGAAGCACGAAAG
Rpl19_Reverse	GCCGCTATGTACAGACACGA
Eef1a1_Forward	AGCTGGCAAAGTCACCAAGT
Eef1a1_Reverse	CCGTTCTTCCACCACTGATT

RNA-sequencing and analysis

Total RNA was purified from frozen NSPC pellets using Trizol and indexed mRNA-seq libraries were constructed and sequenced as described (Collinson *et al.* 2016). Basecalls were converted to fastq using bcl2fastq v2.15.0.4 and read quality was checked using FastQC v0.10.1. Reads were aligned to the mouse genome (GRCm38) using STAR v2.3.0 with default parameters. Where

necessary, subsampling was performed using samtools view with the –s option. Read counts per annotated feature were determined using htseq-count v0.6.0. Gene annotation is taken from Ensembl release 75 and small RNA annotation from miRbase release 20. Raw read counts were further analyses using the BioConductor package limma. Briefly, features with zero reads in all samples were removed and the remaining data were converted to logCPM using the voom function, taking into account mean associated variance and library size. Gene ontology (GO) functional annotation for the identified gene sets was done using the DAVID analysis tool (http://david.abcc.ncifcrf.gov/home.jsp). Corrected *p*-values were calculated using a modified Fisher's exact test followed by Bonferroni's multiple comparison test.

ChIP-sequencing

Cell pellets (from 400,000 cells) were thawed and suspended in 95µl MNase Buffer (50mM Tris-HCl pH8, 1mM CaCl2, 0.2% Triton X-100) supplemented with 5mM Sodium Butyrate and 1X Complete EDTA-free Protease Inhibitor (Roche). Chromatin was digested with micrococcal nuclease (3U; NEB) for 8min at 37°C to obtain predominantly mononucleosomes and dinucleosomes. Stop buffer (10µl of 110mM Tris-HCl pH8, 5mM EDTA) was added to inactivate the reaction. Chromatin was solubilised by brief sonication using a Diagenode Bioruptor for 1min on high power. Chromatin was diluted in 100ml RIPA-IP buffer (280mM NaCl, 1.8% Triton X-100, 0.2% SDS, 0.2% Sodium Deoxycholate, 5mM EGTA supplemented with 5mM Sodium Butyrate and 1X Complete EDTA-free Protease Inhibitor) and insoluble material was removed by centrifugation at 14,000 rpm at 4°C for 15 minutes. Supernatant was transferred to a new tube, 10% was removed for the input sample, and the remaining chromatin was pre-cleared with 50µl pre-washed Protein A and Protein G Dynabeads (Thermo Fisher Scientific) at 4°C for 1 hour. Dynabeads were removed, the pre-cleared chromatin was diluted to 500µl in RIPA-IP buffer and split into 5 tubes. Chromatin was incubated overnight with histone antibodies at 4°C with rotation. Antibodies used were H3K27me3 (Millipore, 07-449; 1µg per ChIP) and H3K4me3 (Abcam,

ab8580; 0.5μg per ChIP). Pre-washed Dynabeads (10μl) were added to each tube and incubated at 4°C for 2-3 hours with rotation. Beads were washed five times with RIPA buffer, once with LiCl buffer (250mM LiCl, 10mM Tris-HCl pH8, 0.5% NP-40, 0.5% Sodium Deoxycholate, 1mM EDTA) and once with 1xTE. Beads were suspended in 100μl 1X TE supplemented with 50μg Proteinase K and incubated at 55°C for 1 hour. ChIP and Input DNA were purified using Genomic DNA Clean and Concentrator columns (Zymo) and eluted in 50μl 1xTE. ChIP material was examined using a small aliquot of material by qPCR. Indexed ChIP-Seq libraries were generated with the NEBNext Master Kit (NEB) using NEBNext Multiplex Oligos for Illumina indexes (NEB). Library fragment size and concentration was determined using an Aglient Bioanalyzer 2100 and KAPA Library Quantification Kit (KAPA Biosystems). Samples were sequenced on an Illumina NextSeq 500 as 75bp single-end libraries.

ChIP-sequencing analysis

Sequencing reads were trimmed using trim galore v0.4 using default parameters to remove the standard Illumina adapter sequence. Reads were mapped to the mouse GRCm38 genome assembly using bowtie2 v2.2.5 using default parameters. BAM files imported to SeqMonk and reads were extended by 200bp at their 5' end to approximate the true insert size. Sequence reads were quantitated over gene promoter regions (defined as -2kb to +0.5kb over transcriptional start sites) and the values were globally normalised per million reads. Gene promoters with log2 RPM values greater than 1, and that differed by more than 1.5-fold in log2 RPM values between adult and aged NSPCs, were defined as differentially methylated. For drawing the quantitation trend plots, sequence reads were quantitated over 100bp running windows, values were globally normalised to the data set with the highest coverage, and used to calculate the mean signal over all transcriptional start sites -/+ 5kb. For the genome browser screenshots, data were quantitated over 20bp running windows, separated by 10bp and the values were globally normalised to the data set with the highest coverage.

BS-sequencing and oxBS-sequencing

Genomic DNA was extracted using the AllPrep DNA/RNA Mini Kit (QIAGEN). DNA (250ng per sample) was fragmented by sonication (Covaris) and spiked with pooled sequencing control samples (3% w/w; CEGX). Samples were end-repaired and ligated with Illumina-supplied methylated adaptors using the NEBnext Ultra Kit (NEB). Indexed BS-seq and oxBS-seq libraries were constructed using the TrueMethyl Seq (CEGX) following the manufacturer's protocol. Final library amplification (11 cycles) was performed with Kapa Uracil Plus (Kapa Biosystems) and purified using 1x XP Ampure beads (Beckman Coulter). Samples were sequenced on an Illumina HiSeq 1000 as 100bp single-end libraries.

Sequence reads were trimmed to remove poor-quality reads and adapter contamination using Trim Galore (v0.3.7). The remaining sequences were mapped using Bismark (v0.12.5) (Krueger & Andrews 2011) with default parameters to the mouse reference genome GRCm38 in paired-end mode. Reads were deduplicated and CpG methylation calls were extracted from the deduplicated mapping output using the Bismark methylation extractor (v0.13.0) in paired-end mode.

Methylation calls were imported into SeqMonk and three replicates were merged into data groups for Adult and Aged, and bisulfite and oxidative bisulfite samples, respectively. Coverage outliers (10-fold above the median) were excluded. The average percentage methylation was calculated over windows generated over 50 consecutive CpGs where at least 10 had been measured in each sample. The percentage methylation of genomic features were calculated in SeqMonk and exported as annotated probe reports. Probes were created over CpG Islands (Illingworth et al. 2008) and probes with at least 10 observations in both data groups were quantified using the bisulfite methylation over features pipeline in SeqMonk. Probes were compared using a Chi-squared test with multiple testing correction, and an adjusted p-value of <0.05 was categorised as significant.

SUPPLEMENTAL REFERENCES

Carucci N, Cacci E, Nisi PS, Licursi V, Paul YL, Biagioni S, Negri R, Rugg-Gunn PJ, Lupo G (2017). Transcriptional response of Hoxb genes to retinoid signalling is regionally restricted along the neural tube rostrocaudal axis. *Royal Society Open Science*. **4**, 160913.

Collinson A, Collier AJ, Morgan NP, Sienerth AR, Chandra T, Andrews S, Rugg-Gunn PJ (2016). Deletion of the polycomb-group protein EZH2 leads to compromised self-renewal and differentiation defects in human embryonic stem cells. *Cell Rep*. **17**, 2700-2714.

De Luca G, Ventura I, Sanghez V, Russo MT, Ajmone-Cat MA, Cacci E, Martire A, Popoli P, Falcone G, Michelini F, Crescenzi M, Degan P, Minghetti L, Bignami M, Calamandrei G (2013). Prolonged lifespan with enhanced exploratory behavior in mice overexpressing the oxidized nucleoside triphosphatase hMTH1. *Aging Cell*. **12**, 695-705.

Esteve P, Sandonis A, Cardozo M, Malapeira J, Ibanez C, Crespo I, Marcos S, Gonzalez-Garcia S, Toribio ML, Arribas J, Shimono A, Guerrero I, Bovolenta P (2011). SFRPs act as negative modulators of ADAM10 to regulate retinal neurogenesis. *Nature Neuro*. **14**, 562-569.

Gong S, Zheng C, Doughty ML, Losos K, Didkovsky N, Schambra UB, Nowak NJ, Joyner A, Leblanc G, Hatten ME, Heintz N (2003). A gene expression atlas of the central nervous system based on bacterial artificial chromosomes. *Nature* **425**, 917–925.

Illingworth R, Kerr A, Desousa D, Jørgensen H, Ellis P, Stalker J, Jackson D, Clee C, Plumb R, Rogers J, Humphray S, Cox T, Langford C, Bird A (2008). A novel CpG island set identifies tissue-specific methylation at developmental gene loci. *PLoS Biol*. **6**, e22.

Krueger F, Andrews SR (2011). Bismark: a flexible aligner and methylation caller for Bisulfite-Seq applications. *Bioinformatics*. **27**, 1571-1572.

Lenzi J, Pagani F, De Santis R, Limatola C, Bozzoni I, Di Angelantonio S, Rosa A (2016). Differentiation of control and ALS mutant human iPSCs into functional skeletal muscle cells, a tool for the study of neuromuscolar diseases. *Stem Cell Res*. **17**, 140-147.

Pratt T, Sharp L, Nichols J, Price DJ, Mason JO (2000). Embryonic stem cells and transgenic mice ubiquitously expressing a tau-tagged green fluorescent protein. *Dev. Biol*. **228**, 19-28.

Soldati C, Cacci E, Biagioni S, Carucci N, Lupo G, Perrone-Capano C, Saggio I, Augusti-Tocco G (2012). Restriction of neural precursor ability to respond to Nurr1 by early regional specification. *PloS One*. **7**, e51798.

Soldati C, Caramanica P, Burney MJ, Toselli C, Bithell A, Augusti-Tocco G, Stanton LW, Biagioni S, Buckley NJ, Cacci E (2015). RE1 silencing transcription factor/neuron-restrictive silencing factor regulates expansion of adult mouse subventricular zone-derived neural stem/progenitor cells in vitro. *J. Neuro Res*. **93**, 1203-1214.

Vallier L, Rugg-Gunn PJ, Bouhon IA, Andersson FK, Sadler AJ, Pedersen RA (2004). Enhancing and diminishing gene function in human embryonic stem cells. *Stem Cells*. **22**, 2-11.
